# Synthesis and Biological Evaluation of RGD–Cryptophycin Conjugates for Targeted Drug Delivery

**DOI:** 10.3390/pharmaceutics11040151

**Published:** 2019-04-01

**Authors:** Adina Borbély, Eduard Figueras, Ana Martins, Simone Esposito, Giulio Auciello, Edith Monteagudo, Annalise Di Marco, Vincenzo Summa, Paola Cordella, Raffaella Perego, Isabell Kemker, Marcel Frese, Paola Gallinari, Christian Steinkühler, Norbert Sewald

**Affiliations:** 1 Organic and Bioorganic Chemistry, Department of Chemistry, Bielefeld University, Universitätsstraße 25, DE-33615 Bielefeld, Germany; adina.borbely@uni-bielefeld.de (A.B.); eduard.figueras@uni-bielefeld.de (E.F.); ana_martins@exiris.it (A.M.); isabell.kemker@uni-bielefeld.de (I.K.); marcel.frese@uni-bielefeld.de (M.F.); 2 Exiris s.r.l., Via di Castel Romano 100, IT-00128 Rome, Italy; paola_gallinari@exiris.it (P.G.); christian_steinkuhler@exiris.it (C.S.); 3 IRBM S.p.A, Via Pontina km. 30,600, IT-00071 Pomezia (Rome), Italy; s.esposito@irbm.com (S.E.); g.auciello@irbm.com (G.A.); e.monteagudo@irbm.com (E.M.); a.dimarco@irbm.com (A.D.M.); v.summa@irbm.com (V.S.); 4 Italfarmaco S.p.A., Via dei Lavoratori, 54, IT-20092 Cinisello Balsamo (Milano), Italy; p.cordella@italfarmaco.com (P.C.); r.perego@italfarmaco.com (R.P.)

**Keywords:** antitumor agents, small molecule–drug conjugates, drug delivery, RGD peptides

## Abstract

Cryptophycins are potent tubulin polymerization inhibitors with picomolar antiproliferative potency in vitro and activity against multidrug-resistant (MDR) cancer cells. Because of neurotoxic side effects and limited efficacy in vivo, cryptophycin-52 failed as a clinical candidate in cancer treatment. However, this class of compounds has emerged as attractive payloads for tumor-targeting applications. In this study, cryptophycin was conjugated to the cyclopeptide *c*(RGDfK), targeting integrin α_v_β_3_, across the protease-cleavable Val-Cit linker and two different self-immolative spacers. Plasma metabolic stability studies in vitro showed that our selected payload displays an improved stability compared to the parent compound, while the stability of the conjugates is strongly influenced by the self-immolative moiety. Cathepsin B cleavage assays revealed that modifications in the linker lead to different drug release profiles. Antiproliferative effects of Arg-Gly-Asp (RGD)–cryptophycin conjugates were evaluated on M21 and M21-L human melanoma cell lines. The low nanomolar in vitro activity of the novel conjugates was associated with inferior selectivity for cell lines with different integrin α_v_β_3_ expression levels. To elucidate the drug delivery process, cryptophycin was replaced by an infrared dye and the obtained conjugates were studied by confocal microscopy.

## 1. Introduction

The selective delivery of cytotoxic drugs to tumors is a promising approach to overcome the drawbacks of traditional chemotherapy, such as limited efficacy and increased systemic toxicity [1]. Several antibody–drug conjugates (ADCs) and small molecule–drug conjugates (SMDCs) have been developed to increase the therapeutic index of cytotoxic agents by conjugating them to a delivery vehicle (i.e., antibody or small molecule) specifically binding to its target antigen present on the tumor cell surface [2,3,4]. Many of them are at various stages of clinical and preclinical development, and four ADCs (Adcetris^®^, Kadcyla^®^, Mylotarg^®^, and Besponsa^®^) have been approved for cancer treatment [5]. Despite the significant success of ADCs, several limitations, like an unfavorable pharmacokinetic profile (low tissue diffusion and low tumor accumulation), immunogenicity, and high manufacturing costs, still need to be addressed [6]. Due to their smaller size, SMDCs have emerged as good alternatives for ADCs. In this case, the straightforward chemical synthesis and easier purification of SMDCs significantly reduces the production costs and allows an extensive hit to lead optimization. Moreover, SMDCs exhibit improved tumor penetration and do not trigger an immune response. However, because of their faster plasma clearance that allows reduced off-target tissue exposure, more frequent dosing may be required [7,8].

**Figure 1:**
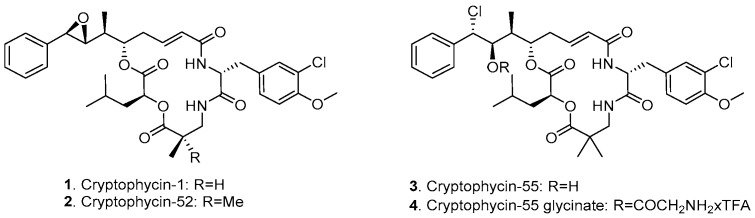
Chemical structure of cryptophycin derivatives.

Cryptophycins are a family of 16-membered macrocyclic depsipeptides, first isolated from cyanobacteria *Nostoc* sp. in 1990 [9]. This class of compounds is able to arrest the cell cycle in the G_2_/M phase by inhibition of microtubule polymerization at substoichiometric concentrations and by depolymerization of microtubules [10,11], resulting in picomolar antiproliferative potency in vitro. Moreover, cryptophycins are poor substrates for P-glycoprotein (P-gp)-mediated transport, leading to retained activity against multidrug-resistant (MDR) cells [12]. The synthetic analogue, cryptophycin-52 (LY355703, **2**, Figure 1) was developed as a potential clinical candidate but failed to exhibit significant response and showed unacceptable levels of neurotoxicity at the administered doses for the treatment of non-small cell lung cancer and platinum-resistant ovarian cancer [13,14]. Further investigations were not only focused on the development of functionalized cryptophycin analogues [15,16,17], but also on the conjugation of cryptophycin to tumor-homing peptides [18,19] and antibodies [20,21,22].

Among the various functionalized cryptophycin derivatives, the chlorohydrin analogue of cryptophycin-52 (cryptophycin-55, **3**, Figure 1) proved to be more active but less stable compared to the parent compound, while the glycinate ester of the chlorohydrin (**4**, Figure 1) displays both chemical stability and comparable or superior in vivo activity against solid tumors of mouse and human origin [23]. The free amino group of the glycinate provides a suitable attachment site for conjugation to delivery vehicles across a carbamate or amide bond formation. We have previously reported the application of cryptophycin-55 glycinate (Cry-55gly) for the development of tumor targeting ADCs [24] and SMDCs [25,26]. The conjugation of this payload to other targeting moieties is of considerable interest.

Integrins, a family of 24 heterodimeric transmembrane glycoproteins composed of 18 different α subunits and 8 β subunits, became widely studied targets in anticancer therapy due to their involvement in many steps of tumor growth [27]. The integrin α_v_β_3_ is highly abundant in several human cancer cells (including melanoma, glioblastoma, prostate, pancreatic, breast cancer); it is overexpressed on the endothelial cells of tumor neovasculature, and it is involved in cancer progression and metastasis [28,29,30]. Integrin α_v_β_3_ recognizes its endogenous ligands by the tripeptide Arg-Gly-Asp (RGD) sequence [31]. Consequently, a large number of linear and cyclic RGD containing peptides and peptidomimetics have been developed with high binding affinity to the receptor [32,33,34]. Cilengitide, *c*(RGDf(*N*Me)V), evolved as a highly active and selective ligand from structure–activity relationship studies utilizing the concept of conformation-dependent recognition. However, it failed in clinical phase III as anti-angiogenic agent for glioblastoma treatment [35]. Based on the fact that replacement of Val to Lys does not affect receptor binding and Lys can be used as a chemical handle to attach different entities, the functionalized cyclic pentapeptide *c*(RGDfK) is broadly applied as carrier for imaging [36,37] and pharmacodelivery [38,39,40,41] applications.

We have previously prepared an azido-functionalized cryptophycin derivative that was conjugated to a modified *c*(RGDfK) peptide across a non-cleavable triazole ring. The resulting conjugate showed an inferior cytotoxicity compared to the parent drug due to the stability of the linkage between the cytotoxin and the RGD ligand which did not allow the release of the free drug. Moreover, we have reported that a fluorescent cryptophycin–RGD conjugate undergoes fast endocytosis and shows colocalization with the lysosomes of human tumor cells [18]. For these reasons, conjugates with lysosomally cleavable linkers represent an excellent potential to ensure increased antitumor activity.

## 2. Materials and Methods

### 2.1. Synthesis of Cryptophycin Conjugates

**Synthesis of Alkynyl-PEG5-Val-Cit-PABC-Cry-55gly (7)**: Cryptophycin-55 glycinate (Cry-55gly, **4**) was synthesized as previously described [25]. Here, **4** (5 mg, 6.5 µmol, 1 eq) and **5** (16 mg, 19.2 µmol, 3 eq) were dissolved in anhydrous *N*,*N*-dimethylformamide (DMF) (500 µL) under argon. *N*,*N*-diisopropylethylamine (DIPEA) (5 µL, 29.2 µmol, 4.5 eq) was added and the mixture was stirred for 4 h at room temperature (RT), followed by reversed-phase high-performance liquid chromatography (RP-HPLC) purification (method M2/b, see Supplementary Material) to yield **7** as a colorless solid (6.0 mg, 63%). **Liquid Chromatography-Mass Spectrometry (LC-MS):** *t*_R_ = 10.28 min, >99% purity (λ = 220 nm), *m*/*z* calcd for [C_71_H_100_Cl_2_N_8_O_20_]^2+^: 727.32 [M + 2H]^2+^, found: 727.34.

**Synthesis of Alkynyl-PEG5-Val-Cit-Gly-Pro-Cry-55gly (8)**: Here, **4** (4 mg, 5.25 µmol, 1 eq), **6** (16 mg, 21 µmol, 4 eq), benzotriazol-1-yloxy)tripyrrolidinophosphonium hexafluorophosphate (PyBOP) (11 mg, 21 µmol, 4 eq), and *N*-hydroxybenzotriazole (HOBt) (4 mg, 24 µmol, 4.5 eq) were dissolved in anhydrous DMF (700 µL) under argon. DIPEA (4 µL, 24 µmol, 4.5 eq) was added and the mixture was stirred for 4 h at RT, followed by RP-HPLC purification (method M2/b) to yield **8** as a colorless solid (6.0 mg, 78%). **LC-MS:** *t*_R_ = 10.05 min, >99% purity (λ = 220 nm), *m*/*z* calcd for [C_70_H_102_Cl_2_N_9_O_20_]^+^: 1458.66 [M + H]^+^, found: 1458.69; *m*/*z* calcd for [C_70_H_103_Cl_2_ N_9_O_20_]^2+^: 729.83 [M + 2H]^2+^, found: 729.85.

**Synthesis of *c*(RGDfK)-PEG5-Val-Cit-PABC-Cry-55gly (10):** Here, **7** (3.5 mg, 2.4 µmol, 1 eq), **9** (1.9 mg, 2.6 µmol, 1.1 eq), CuSO_4_·5H_2_O (0.3 mg, 1.2 µmol, 0.5 eq), and sodium ascorbate (0.29 mg, 1.4 µmol, 0.6 eq) were dissolved in DMF/H_2_O (1:1, 200 µL, degassed) and stirred for 24 h at 35 °C, followed by RP-HPLC purification (method M2/b) to yield **10** as a colorless solid (3.3 mg, 64%). **LC-MS**: *t*_R_ = 7.77 min, >99% purity (λ = 220 nm), *m*/*z* calcd for [C_101_H_144_Cl_2_N_20_O_28_]^2+^: 1077.49 [M + 2H]^2+^, found: 1077.52; *m*/*z* calcd for [C_101_H_145_Cl_2_N_20_O_28_]^3+^: 718.66 [M + 3H]^3+^, found: 718.68. **High Resolution Mass Spectrometry, Electrospray Ionization Mass Spectrometry (HRMS (ESI-MS))**: *m*/*z* calcd for [C_101_H_144_Cl_2_N_20_O_28_]^2+^: 1077.4913 [M + 2H]^2+^, found: 1077.4867; *m*/*z* calcd for [C_101_H_145_Cl_2_N_20_O_28_]^3+^: 718.6633 [M + 3H]^3+^, found: 718.6595.

**Synthesis of *c*(RGDfK)-PEG5-Val-Cit-Gly-Pro-Cry-55gly (11):** Here, **8** (3.5 mg, 2.4 µmol, 1 eq), **9** (1.8 mg, 2.6 µmol, 1.1 eq), CuSO_4_·5H_2_O (0.3 mg, 1.2 µmol, 0.5 eq), and sodium ascorbate (0.29 mg, 1.4 µmol, 0.6 eq) were dissolved in DMF/H_2_O (1:1, 200 µL, degassed) and stirred for 24 h at 35 °C, followed by RP-HPLC purification (method M2/b) to yield **11** as a colorless solid (2.80 mg, 54%). **LC-MS:** *t*_R_ = 7.31 min, >99% purity (λ = 220 nm), *m*/*z* calcd for [C_100_H_147_Cl_2_N_21_O_28_]^2+^: 1080.00 [M + 2H]^2+^, found: 1080.02; *m*/*z* calcd for [C_100_H_148_Cl_2_N_21_O_28_]^3+^: 720.34 [M + 3H]^3+^, found: 720.34; **HRMS (ESI-MS)**: *m*/*z* calcd for [C_100_H_147_Cl_2_N_21_O_28_]^2+^: 1080.0045 [M + 2H]^2+^, found: 1080.0126; *m*/*z* calcd for [C_100_H_148_Cl_2_N_21_O_28_]^3+^: 720.3388 [M + 3H]^3+^, found: 720.3428.

### 2.2. Integrin Binding Assay

An ELISA-like assay using isolated integrins was performed to determine binding affinity [34]. Cilengitide (α_v_β_3_: *IC*_50_ = 0.54 nM) was used as internal reference. All wells of flat-bottom 96-well Immuno Plates (BRAND GmbH & Co. KG, Wertheim, Germany) were coated overnight at 4 °C with 100 µL human vitronectin solution (1.0 µg/mL, #2349-VN-100, R&D Systems, Minneapolis, MI, USA) in carbonate buffer (15 mM Na_2_CO_3_, 35 mM NaHCO_3_, pH 9.6). Each well was then washed with phosphate-buffered saline/Tween20 (PBS-T-buffer, 137 mM NaCl, 2.7 mM KCl, 10 mM Na_2_HPO_4_, 2 mM KH_2_PO_4_, 0.01% Tween20, pH 7.4; 3 × 200 µL) and blocked for 1 h at room temperature with TS-B-buffer (Tris-saline/bovine serum albumin (BSA) buffer, 150 µL/well, 20 mM Tris-HCl, 150 mM NaCl, 1 mM CaCl_2_, 1 mM MgCl_2_, 1 mM MnCl_2_, pH 7.5, 1% BSA). A dilution series of the compound and internal standard were prepared in TS-B-buffer. After washing the assay plate with PBS-T (3 × 200 µL), 50 µL of the dilution series were transferred to each well, for negative control 100 µL TS-B-buffer and for positive control 50 µL TS-B-buffer were used. Then, 50 µL of human α_v_β_3_ integrin (2.0 µg/mL, #3050-AV-050, R&D) in TS-B-buffer were transferred to wells (except wells containing the negative control) and incubated for 1 h at RT. After washing the plates (3 × 200 µL) with PBS-T buffer, 100 µL primary antibody mouse anti-human CD51/61 (2.0 µg/mL, #555504, BD Biosciences, San Jose, CA, USA) were added to all wells and incubated for 1 h at RT. The plate was washed (3 × 200 µL) with PBS-T buffer and 100 µL of secondary, peroxidase linked antibody (anti-mouse IgG-POD goat, 1.0 µg/mL, #A441, Sigma Aldrich, St. Louis, MO, USA) were added to all wells and incubated for 1 h at RT. The plate was washed (3 × 200 µL) with PBS-T buffer and 50 µL SeramunBlau (SeramunBlau fast S-001-1-TMB, Seramun Diagnostica GmbH, Heidesee, Germany) were added to all wells. The development was stopped with 3 M H_2_SO_4_ (50 µL/well) when a blue color gradient was visible (~1 min). The absorbance was measured with a plate reader at 450 nM (Tecan, Infinite M200, Zürich, Switzerland). The resulting curves were analyzed with OriginPro 2017G (OriginLab Corporation, Northampton, MA, USA) with the inflection point describing the *IC*_50_ value. Each compound was tested twice in triplicates and referenced to the internal standard.

### 2.3. Plasma Metabolic Stability Assay

The sample preparation and the metabolic analysis of the conjugates was carried out similarly to a previously described method [42]. In a 96-well plate, the stock solutions (3 mM in dimethyl sulfoxide (DMSO)) of the conjugates and the control compound were diluted with 250 μL of plasma (pooled heparinized mouse ICR/CD1 M plasma #08 or human plasma #MPLLIHP-M, #BHR1003077, BioreclamationIVT, Westbury, NY, USA) to give a 3-µM concentration and incubated at 37 °C. At each sampling time an aliquot of 30 µL was transferred into a 96-deep-well plate and the reaction was stopped with 120 µL of acetonitrile (ACN) containing 0.1% HCOOH and 400 ng/mL of warfarin as internal standard (IS). This mixture was centrifuged at 1100× *g* for 30 min at 4 °C and 50 µL of supernatants were transferred into a clean 96-deep-well plate and diluted with 50 µL of 0.1% HCOOH in water. Samples were stored at −80 °C prior to UPLC-HRMS analysis (see Supplementary Materials). The stability was determined based on the disappearance of the test compound as a function of incubation time, using area ratios (analyte peak area vs internal standard peak area). The elimination constant k was calculated by plotting mean disappearance values on a semi-logarithmic scale and fitting with a best fit linear regression using Microsoft Excel. The half-life (*t*_1/2_) expressed in hours was determined using equation: *t*_1/2_ = ln2/(−*k*). Full metabolite analysis was done using Compound Discoverer^TM^ 2.1 (Thermo Fisher Scientific, Waltham, MA, USA) software.

### 2.4. Cathepsin B Cleavage Assay

l-cysteine (#C8152, Sigma Aldrich) was dissolved in acetate buffer/ethylenediaminetetraacetic acid (EDTA) 1 mM pH 5.5 to get a solution with a final concentration of l-cysteine of 28 mM. This solution was used to dilute an aliquot of cathepsin B (bovine spleen cathepsin B, #C6286, Sigma Aldrich) to 1.11 U/mL. Sample containing cathepsin B was pre-incubated at 37 °C for 15 min and then split into 45-µL aliquots in Eppendorf tubes, two replicates for each incubation time. Aliquots of 5 µL of 50 µM solutions of the substrates prepared in MeOH/H_2_O (50/50) were added to each tube, to get a final concentration of 5 µM. Tubes corresponding to *t* = 0 min contained 100 µL of 1% HCOOH in MeOH and were put in an ice bath to stop the reaction. All the other tubes were incubated at 37 °C in an oscillating thermostatic bath and reaction was stopped at the following incubation times: 0.25, 0.5, 1, 2, 4, and 6 h, as described for *t* = 0 min samples. Samples were centrifuged for 10 min at 14,000 rpm at 4 °C and filtered with regenerated cellulose syringe filters prior to injection in the HPLC-MS system (see Supplementary Materials). Control solutions of each compound (5 µM in acetate buffer pH 5.5/EDTA 1 mM/l-cysteine 28 mM) were also prepared and incubated up to 6 h at 37 °C in the absence of the enzyme to check substrate stability at the incubation conditions. Substrates and possible metabolites were quantified based on calibration curves prepared in acetate buffer/EDTA/l-cysteine.

### 2.5. Degradation in Human Liver Lysosomal Homogenate

Pooled human liver lysosomes (#H0610.L) at 2.5 mg protein/mL, cathepsin B activity 96.5 ± 3.96 units/mg protein were purchased from Sekisui XenoTech (Kansas City, KS, USA). 

Experiments were performed based on the method described previously [43], with some modifications. Conjugates and control compound (Bz-FVR-*p*-nitroanilide, #B7632, Sigma Aldrich) were diluted in 0.2 M citrate phosphate buffer, pH 5.5, containing 0.1% (*v*/*v*%) Triton X-100, 5 mM reduced glutathione, 1 mM EDTA, and human liver lysosomes (1.5 mg protein/mL) to give a 3 µM final concentration. At each time point (0, 0.5, 1, and 2 h) an aliquot of 30 μL was transferred into a new 96-deep-well plate and the reaction was stopped with 120 μL of ACN containing 0.1% HCOOH and 0.4 µg/mL of warfarin as internal standard (IS). At the end of the assay, the plate was centrifuged at 1100× *g* for 30 min at 4 °C and 50 μL of the supernatant were transferred into a new 96-deep-well plate. The sample was diluted with 50 μL of mixture of ACN/H_2_O/HCOOH = 20/80/0.1. Samples were stored at −80 °C prior to UPLC-HRMS analysis (see Supplementary Materials). 

### 2.6. In Vitro Cytotoxicity Assay

The human melanoma M21 and M21-L cells were kindly provided by Dr. David Cheresh and The Scripps Research Institute (La Jolla, CA, USA). Cells were cultured in RPMI 1640 supplemented with 10% fetal bovine serum (FBS), 1% l-glutamine and 1% Penicillin/Streptomycin in a humidified incubator at 37 °C and 5% CO_2_.

Cell viability was quantified by 3-(4,5-dimethylthiazol-2-yl)-2,5-diphenyltetrazolium bromide (MTT) assay. Cells were plated at a density of 3000 cells/well (Nunc^®^ MicroWell^®^ flat-bottom 96-well plate, Thermo Fisher Scientific) and incubated overnight to allow adherence. The following day, cells were treated with serial dilutions of each compound or 0.1% DMSO as control. After 2 h of incubation, cells were centrifuged at 1200 rpm for 5 min; supernatant containing the compounds was removed and replaced with fresh medium. Cells were further incubated for 70 h as described above, and at the end of treatment, 5 µL of MTT (5 mg/mL in deionized H_2_O, #M5655, Sigma Aldrich) were added to each well and the cells incubated for another 2 h. Finally, 100 µL of lysis buffer (10% SDS, 10 mM HCl) were added, and cells were placed in the incubator overnight for the formazan crystal solubilization. Absorbance at 540 nm was measured using FLUOstar Omega (BMG Labtech, Ortenberg, Germany) instrument and the growth inhibition ratio was calculated. Blank controls detecting cell-free media absorbance were performed in parallel. Three experimental replicates were used. The half-maximal inhibitory concentration values (*IC*_50_) were obtained from viability curves by fitting data to the four-parameter logistic equation using GraphPad Prism 6 (GraphPad Software, San Diego, CA, USA). The cell viability was expressed as percentage relative to the respective control conditions. 

### 2.7. Flow Cytometry Analysis

The M21 and M21-L cells were plated at a density of 80,000 cells/mL/well in a 12-well plate and incubated overnight at 37 °C. The next day, cells were incubated with LM142 mouse anti-human integrin α_V_ monoclonal antibody (#MAB1978, Merck Millipore, Merck KGaA, Darmstadt, Germany, 1:500 dilution) or with mouse anti-human CD51/CD61 (#555504, BD Biosciences, San Jose, CA, USA) at 1 µg/mL for 15 min on ice. Subsequently, cells were washed with PBS and incubated with anti-mouse IgG-Alexa Fluor 488 (#A-11029, Thermo Fisher Scientific) at 8.3 µg/mL for 15 min on ice. Finally, cells were washed with PBS, and samples were acquired using BD FACSCalibur^TM^ flow cytometer and analyzed using FlowJo software v.10 (FlowJo LCC, Ashland, OR, USA). Anti-mouse IgG antibody was used as the negative isotope control.

### 2.8. Confocal Microscopy

The real-time internalization of the fluorescently labeled compounds **15** and **16** was acquired using a ZEISS LSM 880 confocal microscope (Carl Zeiss AG, Oberkochen, Germany) coupled with an environmental chamber allowing a 37 °C, 5% CO_2_ atmosphere. The M21 and M21-L human melanoma cells were grown overnight in eight-well chambered slides (Nunc^®^ Lab-Tek^®^, Sigma Aldrich) previously coated with poly-d-Lysine (10 µg/mL, Sigma Aldrich). The fluorescently labeled conjugates were diluted in phenol red-free media to 1 µM (0.1% DMSO) and warmed up to 37 °C. Picture acquisition was made using a 40× water immersion objective and started shortly after the solutions were added to the cells, a frame was made every 30 s for a total of 60 frames. Brightfield was used to distinguish the cell’s structures.

## 3. Results and Discussion

### 3.1. Design and Synthesis

The RGD–cryptophycin conjugates consist of the highly cytotoxic payload, cryptophycin-55 glycinate, and *c*(RGDfK) peptide connected through the protease-cleavable Val-Cit dipeptide linker, which is extensively used in the ADC field [44]. This peptide-based linker displays an excellent balance between high stability in circulation and rapid intracellular cleavage in the presence of lysosomal proteases, mainly cathepsin B [45] and other cysteine cathepsins [46,47]. In order to achieve efficient release of the cytotoxin in an active form, two different self-immolative spacers were inserted between the drug and the cleavage site: the *para*-aminobenzyloxycarbonyl (PABC) spacer, which undergoes 1,6-elimination, or the Gly-Pro dipeptide unit, which was designed to decompose by diketopiperazine formation [48,49]. A polyethylene glycol (PEG5) spacer was introduced between the integrin ligand and the cleavage site to improve water solubility of the conjugate.

**Scheme 1:**
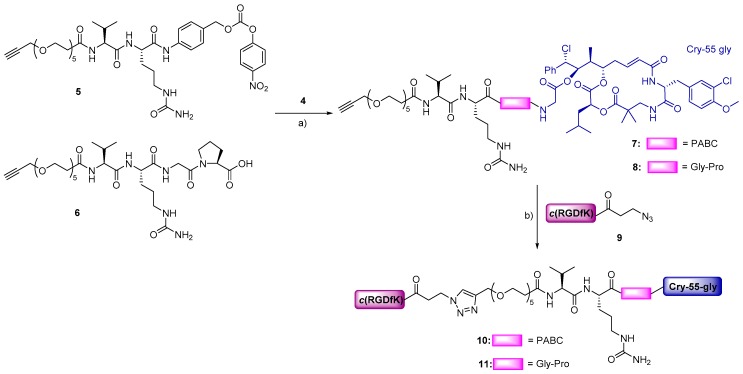
Synthesis of conjugates **10** and **11**. Reagents and conditions: (a) **5** (1.1 eq), DIPEA (3 eq), DMF, RT, 5 h or **6** (3 eq), PyBOP (3 eq), HOBt (3.5 eq), DIPEA (3.5 eq), DMF, RT, 5 h; (b) **9** (1.1 eq), CuSO_4_·5H_2_O (0.5 eq), sodium ascorbate (0.6 eq), 1:1 DMF/H_2_O, 35 °C, 24 h.

The RGD–cryptophycin conjugates **10** and **11** were prepared as shown in Scheme 1. For the synthesis of cleavable linker **5**, Val-Cit-PABOH was reacted with an alkyne-functionalized polyethylene glycol spacer (**19**), followed by activation with bis(4-nitrophenyl) carbonate giving the desired compound (Scheme S2). The cleavable linker **6** was synthetized by standard Fmoc/*^t^*Bu solid-phase peptide synthesis (SPPS) on 2-chlorotrityl chloride resin. The Val-Cit-Gly-Pro tetrapeptide was elongated by the coupling of polyethylene glycol spacer (**19**) containing alkyne moiety on the solid support. After cleavage from the resin and purification by preparative HPLC, the peptide linker was obtained in good yield (32%) and excellent purity. Linkers **5** and **6** were conjugated to cryptophycin-55 glycinate (**4**), which was synthetized as described previously [25], affording the corresponding linker-drugs **7** and **8** in good yields (63–78%). The lysine residue of *c*(RGDfK) peptide was modified with 3-azidopropanoic acid allowing the conjugation of the ligand **9** to the linker-drugs **7** and **8** by Cu(I) catalyzed azide–alkyne cycloaddition (CuAAC click reaction). The resulting final conjugates **10** and **11** were purified by preparative HPLC and characterized by analytical HPLC and HRMS.

### 3.2. Integrin Binding Affinity

**Figure 2:**
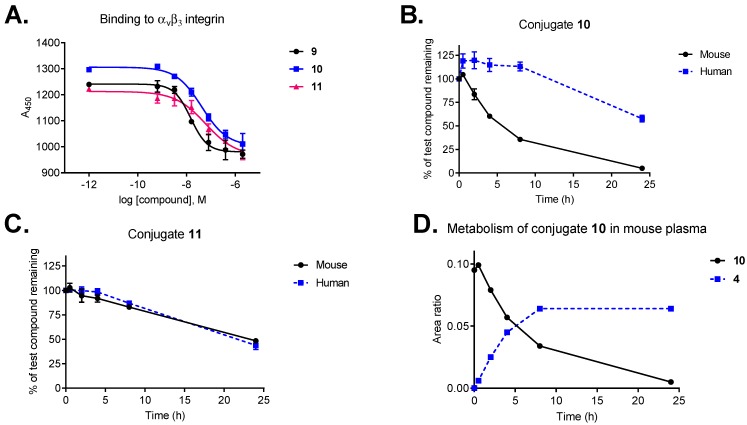
(**A**) Integrin α_v_β_3_ binding affinity of conjugates **10** and **11** compared to the free ligand (**9**). (**B**) Plasma stability of conjugate **10** in mouse and human plasma. (**C**) Plasma stability of conjugate **11** in mouse and human plasma. (**D**) Metabolism of conjugate **10** in mouse plasma and the release of cryptophycin-55 glycinate (**4**). Data points are averages of three experiments, error bars indicate SD.

To investigate how the conjugation affects the binding properties of our ligand, the binding affinity of conjugates **10** and **11** was compared to those of free ligands (**9** and cilengitide) using a competitive ELISA-based assay (Figure 2A). Integrin binding assays were performed by incubation of immobilized vitronectin with increasing concentrations of the test compound (10^−5^–10^−12^ M) in the presence of soluble human α_v_β_3_ integrin. Functionalization of ligand **9** has a negligible effect on receptor binding (*IC*_50_: 0.71 nM vs cilengitide *IC*_50_: 0.54 nM) [34]. Despite the increased size and steric bulkiness, conjugates **10** and **11** retained strong binding affinity to the receptor with *IC*_50_ values in the low nanomolar range (8.3 nM and 12.7 nM, respectively).

### 3.3. Plasma Metabolic Stability

Keeping in mind that our conjugates contain moieties susceptible for hydrolysis, and in vivo payload deactivating metabolism of antibody–cryptophycin conjugates was also reported [50], the in vitro plasma metabolic stability of cryptophycins and RGD–cryptophycin conjugates was assayed in human and mouse plasma.

Plasma metabolic stability studies demonstrated that our selected payload (cryptophycin-55 glycinate, **4**) shows markedly improved stability compared to the parent compound (cryptophycin-52, **2**) in murine plasma and a good stability in human plasma over 24 h (Figure S1A,B). Cryptophycin-52 demonstrated significant metabolism in mouse plasma, including hydrolysis of the macrocycle and loss of units C and D (Figure S1C,D). The same metabolic pathway was proposed for an epoxide containing cryptophycin analog used as ADC payload [50]. In contrast, esterification of the chlorohydrin derivative with glycine was found to provide an enhanced protection of the macrocycle. Taking into account, that cryptophycin-55 glycinate was not affected by ester or amide hydrolysis, increases its potential to be an effective payload.

Conjugates **10** and **11** display good stability in human plasma, with half-lives of more than 24 h and 20 h, respectively (Figure 2B,C). Interestingly, stability in mouse plasma was influenced by the type of self-immolative moiety, conjugate **11** containing the peptide-based Val-Cit-Gly-Pro linker displayed superior stability (*t*_1/2_ = 23 h) compared to conjugate **10** with the Val-Cit-PABC linker (*t*_1/2_ = 6 h). Metabolite identification revealed that the most relevant biotransformation, responsible for the lack of stability of conjugate **10** in mouse plasma, is the hydrolysis of the Val-Cit-PABC linker, resulting in the release of cryptophycin-55 glycinate as major metabolite (Figure 2D). The amide bond between the Cit and the PABC self-immolative moiety is known to be susceptible to enzymatic hydrolysis in mouse plasma [51]. Full metabolite analysis revealed additional minor metabolites of conjugate **10** in mouse plasma (Table S1, Figure S2).

Our results are consistent with previously published data, showing good stability of Val-Cit-PABC-containing conjugates in human plasma [45] and comparable mouse serum stability of SMDCs bearing the same linker [52]. Furthermore, conjugate **11** containing the Val-Cit-Gly-Pro peptidic linker has similar plasma stability as an octreotide–cryptophycin conjugate having the same linker [26].

### 3.4. Drug Release by Cathepsin B and Degradation in Human Liver Lysosomes

**Figure 3:**
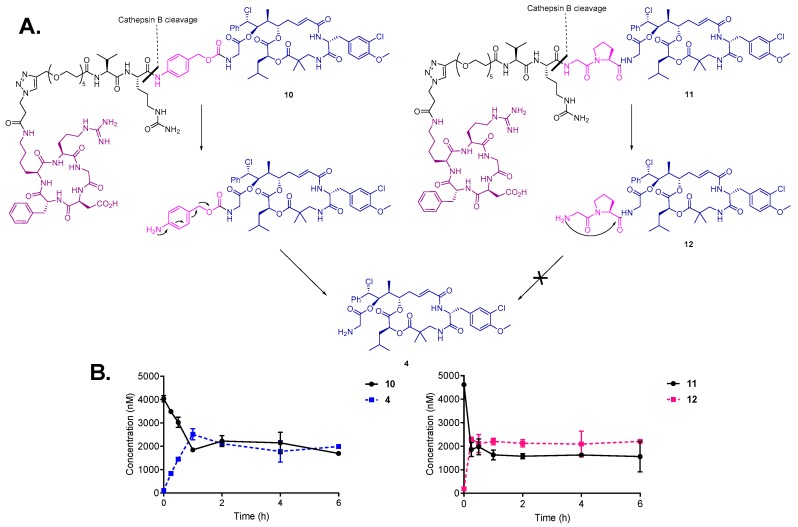
(**A**) Cleavage and self-immolation mechanism of conjugates **10** and **11** in the presence of cathepsin B enzyme. (**B**) Cleavage and metabolism of conjugate **10** (left) and **11** (right) upon incubation with cathepsin B enzyme.

To confirm the susceptibility of the bioconjugates to enzymatic cleavage and prove efficient drug release, the cleavage of conjugates **10** and **11** was monitored in the presence of cathepsin B (1 U/mL) at 37 °C. As expected, conjugates **10** and **11** were rapidly cleaved upon incubation with the enzyme (Figure 3B). In case of conjugate **10**, the enzymatic cleavage of Val-Cit linker resulted in 1,6-elimination of the PABC-spacer and the release of the highly active payload cryptophycin-55 glycinate (**4**) was observed within 15 min following incubation. These results are in agreement with previously reported studies of enzymatic drug release from SMDCs and ADCs containing the Val-Cit-PABC linker [45,53]. In contrast, the cathepsin B mediated linker cleavage of conjugate **11** led to the formation of Gly-Pro-Cry-55gly (**12**), which was not further metabolized to cryptophycin-55 glycinate under the test conditions. This indicates that the enzymatic activation is not followed by intramolecular cyclization and self-immolation of the Gly-Pro dipeptide (Figure 3A).

Although it was reported that prodrugs that contain the Gly-Pro dipeptide linked to paclitaxel via ester bond are able to efficiently release paclitaxel after incubation with plasmin for 3 h [54], our results indicate that self-immolation by diketopiperazine formation does not take place when the Gly-Pro dipeptide is connected to the drug via amide bond. However, the cytotoxicity of metabolite **12** is still of high relevance for the biological activity of conjugate **11**.

In a similar experiment, incubation of conjugates **10** and **11** with human liver lysosomes confirmed the above presented metabolic pathway for each conjugate. Cryptophycin-55 glycinate was found to be released in the case of conjugate **10**, while the formation of Gly-Pro-Cry-55gly metabolite was characteristic for conjugate **11**, without further self-immolation step (Figures S3 and S4). Control experiments indicated that both conjugates are stable in the absence of enzyme or lysosomes. These experiments demonstrated efficient linker processing in the presence of cathepsin B and in the intracellular compartment. Additionally, the character of self-immolative spacer resulted in different drug release profiles.

### 3.5. In Vitro Cytotoxicity Assay

**Figure 4:**
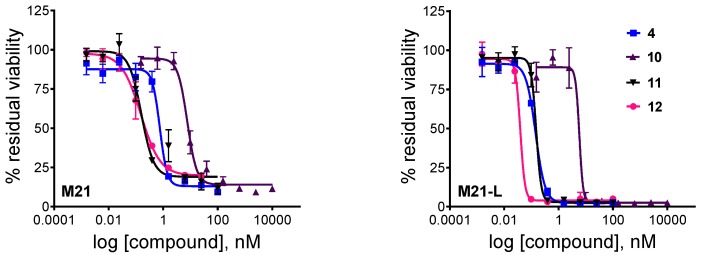
Cytotoxic effect of unconjugated cryptophycin-55 glycinate (**4**), conjugates **10**, **11,** and metabolite **12** against M21 and M21-L human melanoma cells after 72 h (2 h treatment and additional 70 h incubation). Curves obtained by non-linear regression (four-parameter dose-response, error bars represent the standard deviation of triplicates, the measurements were repeated twice).

Having determined that the self-immolative moiety has a great impact on the intracellular activation of the RGD–cryptophycin conjugates, an MTT-based cell viability assay was used to evaluate the in vitro cytotoxicity of the payload **4**, the drug-containing metabolite **12**, and conjugates **10** and **11** (Table 1, Figure 4). The M21 and M21-L human melanoma cells were used to test in vitro the tumor-targeting potential of the novel conjugates [55,56]. The M21 cells overexpress α_v_β_3_ integrin, while the M21-L, a stable variant of the wild type cell line, fails to express integrin α_v_, thus causing a lack of α_v_ integrin heterodimers (α_v_β_3_, α_v_β_5_, and α_v_β_6_). The integrin α_v_ and α_v_β_3_ expression was confirmed by flow cytometry analysis (Figure S5).

Cryptophycin-55 glycinate displayed high in vitro potency against both cell lines with *IC*_50_ values in the sub nanomolar range. However, the parent compound, cryptophycin-52, showed a 10-20-fold higher biological activity using the same cell lines and test conditions (*IC*_50_ = 0.07 nM and 0.02 nM in M21 and M21-L cells, respectively). Furthermore, the Gly-Pro-Cry-55gly (**12**) metabolite was similarly active as the free payload, suggesting that inefficient drug release does not compromise biological activity. These data demonstrate that esterification of the chlorohydrin with glycine or Gly-Pro-Gly tripeptide may result only in a slightly decreased potency compared to cryptophycin-52, likely due to different membrane permeability or lower tubulin binding affinity of these derivatives.

Both conjugates are highly potent; the determined *IC*_50_ values are within a range of 0.15–7.63 nM. Conjugate **10** containing the Val-Cit-PABC linker shows slightly reduced activity compared to cryptophycin-55 glycinate, while conjugate **11** bearing the Val-Cit-Gly-Pro linker shows equal potency to that of metabolite **12**. Moreover, conjugate **11** was ~300-fold more potent compared to the model compound azido-functionalized cryptophycin-RGD conjugate [18], verifying the potential of cryptophycin-based conjugates containing cleavable linkers.

Both conjugates exhibit similar cytotoxicity against the antigen-positive and antigen-negative cell line, indicating that there is no apparent correlation between the integrin α_v_/α_v_β_3_ expression level and the observed in vitro potency. It has been shown, similarly, that cytotoxicity of the RGD peptidomimetic-α–amanitin conjugates was not integrin-specific when tested on human glioblastoma U-87 (α_v_β_3_+) and breast adenocarcinoma MDA-MB-468 (α_v_β_3_-) cells, and it was then proposed that the internalization process was mediated by integrins different from α_v_β_3_ (e.g., α_v_β_5_) [57]. To better understand these findings, confocal microscopy studies were performed to investigate the delivery and uptake of RGD-conjugates in M21 and M21-L human melanoma cells.

### 3.6. Intracellular Uptake Studies

To explore cell surface binding and delivery of RGD conjugates, two analogous conjugates were synthetized, in which the cytotoxic payload was replaced with the infrared dye, cyanine5.5. Self-immolative linkers **5** and **6** were reacted with the Cy5.5 amine in good yields giving compounds **13** and **14** (Scheme S4). Fluorescently labeled conjugates **15** and **16** were obtained in satisfactory yields and excellent purity by CuAAC click reaction with the azido-functionalized RGD-ligand **9**. Confocal microscopy studies were carried out with living M21 and M21-L cells. Similar binding and intracellular uptake could be observed for the two fluorescently labeled compounds in both cell lines after a period of 30 min (Figure S6). The intracellular uptake of compounds **15** and **16** in the M21-L cells indicates that internalization of these is mediated by a process independent of α_v_-integrin. The results, applying constructs of *c*(RGDfK)-linker-fluorescent dye, are consistent with previous reports showing that endocytosis of *cyclo*-RGD peptides is integrin independent and follows a fluid phase uptake [58]. In a different study, an α_v_β_3_-independent uptake was confirmed for the monomeric RGD-peptide, while the multimeric RAFT-RGD-Cy5 was efficiently internalized with integrin α_v_β_3_ via clathrin-mediated endocytosis [59]. All these findings support the explanation that a non-integrin-mediated uptake of the cryptophycin-conjugates **10** and **11** can be accounted for the observed in vitro potencies.

Apart from the involvement of the targeting RGD-ligand for the internalization of the SMDCs, the high hydrophobicity of the cytotoxic payload may significantly accelerate the uptake of the RGD–cryptophycin conjugates, and thus compromise the in vitro selectivity. In fact, it was shown that the uptake of ^3^H-cryptophycin-52 by THP-1 and H-125 cells was rapid, reaching a plateau within 20 min, and irreversible, as no dissociation or efflux from the cells was observed [60]. In a separate experiment, ^3^H-cryptophycin-52 became 730-fold more concentrated in HeLa cells upon 20 h incubation [61]. Although the short treatment time in the cytotoxicity assay aimed to minimize such undesired interactions, a nonspecific endocytic process mediated by the hydrophobic payload, that promotes internalization of the conjugates in the antigen-negative cells, cannot be excluded.

## 4. Conclusions

In summary, we have described the synthesis of two novel integrin-targeted SMDCs based on the conjugation of the highly potent cytotoxic payload, cryptophycin-55 glycinate, to the *c*(RGDfK) integrin ligand through the protease sensible Val-Cit dipeptide linker combined with the PABC or Gly-Pro self-immolative moieties. First, it was shown that our selected payload is remarkably more stable in mouse plasma compared to the parent compound, cryptophycin-52, and it is not affected by ester or amide hydrolysis. The exceptional stability of cryptophycin-55 glycinate verifies its development as SMDC payload. Secondly, it was found that the biological and biochemical properties of the conjugates strongly depend on the linker type. On one hand, conjugate **10** bearing the Val-Cit-PABC linker exhibited satisfactory stability in mouse plasma, and it was highly stable in human plasma. Furthermore, its linker was efficiently cleaved in the presence of cathepsin B releasing free cryptophycin-55 glycinate. The conjugate displayed high in vitro potency against M21 and M21-L human melanoma cells with *IC*_50_ values in the low nanomolar range. On the other hand, conjugate **11** with the Val-Cit-Gly-Pro peptide linker showed excellent stability both in mouse and human plasma. Enzymatic linker cleavage resulted in the release of the Gly-Pro-Cry-55gly (**12**) metabolite, indicating the lack of an efficient self-immolation step. Despite the inefficient release of the free drug, conjugate **11** showed enhanced potency compared to conjugate **10**, with *IC*_50_ values in the sub nanomolar range.

Finally, the excellent in vitro activity of the novel bioconjugates was accompanied with inferior selectivity for cell lines with different integrin α_v_β_3_ expression level. These results indicate that the RGD–cryptophycin conjugates are internalized by a nonspecific process, which can be attributed both to the RGD ligand and the high hydrophobicity of the payload and/or conjugates. In the light of these results, further opportunities arise for the design of novel cryptophycin-based therapeutic agents with improved tumor selectivity.

## Figures and Tables

**Table 1:** Cytotoxic potencies of cryptophycin-55 glycinate (**4**), drug-containing metabolite **12**, and bioconjugates **10** and **11** against M21 and M21-L human melanoma cells.

Entry	Compound	*IC*_50_ (nM)	
		M21 (α_v_β_3_+)	M21-L (α_v_-, α_v_β_3_-)
1	Cry-55gly (**4**)	0.75 ± 0.11	0.14 ± 0.08
2	Gly-Pro-Cry-55gly (**12**)	0.15 ± 0.03	0.039 ± 0.01
3	*c*(RGDfK)-Val-Cit-PABC-Cry-55gly (**10**)	7.63 ± 0.76	5.80 ± 4.30
4	*c*(RGDfK)-Val-Cit-Gly-Pro-Cry-55gly (**11**)	0.16 ± 0.02	0.15 ± 0.02

## Supplementary Materials

The following are available online at https://www.mdpi.com/1999-4923/11/4/151/s1: General information, Synthetic procedures: Synthesis of alkyne-functionalized PEG5-linker (**19**), Synthesis of alkynyl-PEG5-Val-Cit-PABC-PNP linker (**5**), Synthesis of alkynyl-PEG5-Val-Cit-Gly-Pro linker (**6**), Synthesis of azido-functionalized RGD-ligand (**9**), Synthesis of Cy5.5 labeled conjugates **15** and **16**, Characterization details for compounds **5**–**23**. Figure S1: Plasma stability of cryptophycin-52 (**A**) and cryptophycin-55 glycinate (**B**). Metabolism of cryptophycin-52 in mouse plasma (**C**) and the structure of the detected metabolites (**D**), Table S1: Major metabolites of **10** identified after 24 h incubation with mouse and human plasma: proposed structure of metabolites, including also their chemical formula, *m*/*z*, retention time (RT), and occurrence (ND: not detected); Figure S2: Plot of the compound **10** and metabolites vs. incubation time (h), Figure S3: Degradation of conjugate **10** by incubation with lysosomal homogenate for 2 h and the release of cryptophycin-55 glycinate (**4**), Figure S4: Degradation of conjugate **11** by incubation with lysosomal homogenate for 2 h and the release of Gly-Pro-Cry-55gly (**12**), Figure S5: Flow cytometry analysis of integrin α_V_ and α_V_β_3_ expression in M21 and M21-L cell lines, Figure S6: Binding and internalization of compounds **15** and **16** (1 µM) in live M21 and M21-L human melanoma cells after incubation at 37 °C for 30 min. Scheme S1: Synthesis of alkyne-functionalized PEG5-linker (**19**), Scheme S2: Synthesis of Alkynyl-PEG5-Val-Cit-PABC-PNP (**5**), Scheme S3: Synthesis of azido-functionalized RGD-ligand (**9**), Scheme S4: Synthesis of conjugates **15** and **16**.
