# Supplementary material for: Synthesis and Biological Evaluation of RGD–Cryptophycin Conjugates for Targeted Drug Delivery
Source: Pharmaceutics. 2019 Apr 1;11(4):151. doi: 10.3390/pharmaceutics11040151 (PMC6523311; doi:10.3390/pharmaceutics11040151)
Supplement: Supplementary file 1 [file pharmaceutics-11-00151-s001.pdf]

Supporting information

for

Synthesis and Biological Evaluation of RGD–Cryptophycin Conjugates for Targeted Drug Delivery

Adina Borbély,[1] Eduard Figueras,[1] Ana Martins,[1,2] Simone Esposito,[3] Giulio Auciello,[3] Edith Monteagudo,[3] Annalise Di Marco,[3] Vincenzo Summa,[3] Paola Cordella,[4] Raffaella Perego,[4] Isabell Kemker,[1] Marcel Frese,[1] Paola Gallinari,[2] Christian Steinkühler,[2,4] and Norbert Sewald[1*]

[1] Organic and Bioorganic Chemistry, Department of Chemistry, Bielefeld University, Universitätsstraße 25, DE-33615 Bielefeld, Germany

[2] Exiris s.r.l., Via di Castel Romano 100, IT-00128 Rome, Italy

[3] IRBM S.p.A, Via Pontina km. 30,600, IT-00071 Pomezia (RM), Italy

[4] Italfarmaco S.p.A., Via dei Lavoratori, 54, IT-20092 Cinisello Balsamo (MI), Italy

*Email: norbert.sewald@uni-bielefeld.de

Table of Contents

General information 3

Synthetic procedures 6

- **Synthesis of alkyne-functionalized PEG5-linker (19)** 6
- **Synthesis of Alkynyl-PEG5-Val-Cit-PABC-PNP linker (5)** 7
- **Synthesis of Alkynyl-PEG5-Val-Cit-Gly-Pro linker (6)** 9
- **Synthesis of azido-functionalized RGD-ligand (9)** 10
- **Synthesis of Cy5.5 labeled conjugates 15 and 16** 13

Characterization details (^{1}H-NMR, HPLC and Mass Spectrometry Data) 16

Supplementary figures 33

References 37

General information

All experiments requiring anhydrous conditions were performed using oven-dried glassware under argon atmosphere. DCM was distilled from CaH_2, THF was distilled from sodium/benzophenone, DMF was dried over 4Å molecular sieves. Chemicals and solvents (reagent-grade or analytical-grade) were purchased from commercial sources and used without further purification. Macherey-Nagel silica gel "Kieselgel 60" with 40-63 µM (230-400 mesh) was used as stationary phase for flash chromatography. Reactions were monitored by TLC (Merck Kieselgel 60, F254 on aluminium foil), spots were visualized with UV-light or by staining with potassium permanganate or cerium molybdate solution.

High Performance Liquid Chromatography - Mass Spectrometry

Analytical HPLC-MS was performed using an Agilent 1200 series consisting of an autosampler, degasser, binary pump, column oven and diode array detector coupled to an Agilent 6220 accurate-mass TOF-MS, equipped with Phenomenex Luna® 3 C18(2) 100 Å (100 mm × 2 mm, 3 µm) column. Analyses were performed in positive ion mode.

Eluent A: H_2O/ACN/HCOOH = 95/5/0.1 and eluent B: H_2O/ACN/HCOOH = 5/95/0.1.

Method M1:

Flow rate: 300 µL/min

0 min	100% A	0% B
10 min	2% A	98% B
11 min	2% A	98% B
11.5 min	100% A	0% B
15 min	100% A	0% B

High resolution mass spectra (HRMS) were recorded on Agilent 6200 accurate mass TOF MS. Samples were injected through an Agilent 1200 LC system, Hypersil Gold C18 (50 mm × 2.1 mm, 1.9 µm) column and linear gradient from 0% to 98 % B at 250 µL/min over 4 minutes, same solvents as before. External calibration, using Agilent tuning mix, was performed before measurements.

Semi-preparative and preparative RP-HPLC was performed on a Merck-Hitachi system (controller: D-7000, pump: L7150, detector: L7420, UV-absorption measured at λ=220 nm), equipped with preparative column: Macherey-Nagel Nucleosil 100-10 C18, 10 µm, 250 mm x

21 mm, M2/a, or semi-preparative column: Macherey-Nagel Nucleosil 100-7 C18, 7 µm, 250 mm × 10 mm, M2/b.

Eluent A: H_2O/ACN/TFA = 95/5/0.1 and eluent B: H_2O/ACN/TFA = 5/95/0.1

Method M2:

Flow rate: 10 mL/min (a) or 4 mL/min (b)

0 min	100% A	0% B
5 min	100% A	0% B
35 min	0% A	100% B
40 min	0% A	100% B
45 min	100% A	0% B

HPLC-MS conditions for Cathepsin B cleavage studies

Samples were analyzed using a HPLC (Prominence, Shimadzu) connected to a triple quadrupole mass spectrometer (API4000, Sciex). A Jupiter C18 300 Å (50 mm × 2 mm) 5 µm particle size was used as a column.

Eluent A: H_2O/ACN/HCOOH = 90/10/0.1 and eluent B: ACN/HCOOH 99.9/0.1.

Flow rate: 200 µL/min

0 min	60% A	40% B
5 min	60% A	40% B
5.1 min	0% A	100% B
7 min	0% A	100% B
7.1 min	60% A	40% B
10 min	60% A	40% B

UPLC-HRMS conditions for plasma stability and lysosomal degradation assays

Samples were analysed on a system consisting of Dionex Ultimate 3000 RS Pump coupled with (a) Dionex Ultimate 3000 RS from Thermo Scientific (Bremen, Germany) autosampler or (b) PAL LSI from CTC Analytics AG (Zwingen, Switzerland) autosampler. UPLC Peptide BEH C18 (50 mm × 2.1 mm, 1.7 µm, 130 Å) column from Waters (Wexford, Ireland) at 40 °C was used for chromatographic separation. A volume of (a) 2 µL or (b) 5 µL was injected.

Eluent A: H_2O/HCOOH = 99.9/0.1 and eluent B: ACN/HCOOH 99.9/0.1.

Flow rate: 400 µL/min

0 min	99.5% A	0.5% B
4.0 min	5% A	95% B
5.0 min	5% A	95% B
5.1 min	99.5% A	0.5% B
6.0 min	99.5% A	0.5% B

All analyses were performed on a Q-Exactive Orbitrap™ mass spectrometer (Thermo Scientific) in ESI positive full scan/data-dependent MS/MS (FS-dd-MS/MS). Each cycle contains four scan events: Full Scan with m/z range (a) 150–1600 or (b) 200–2000 and resolution 35,000 FWHM at 200 m/z, mass accuracy: 5 ppm, followed by three MS/MS fragmentation scans with resolution 17,500 FWHM at 200 m/z over the three most abundant ions (Top N = 3) of the full-MS spectrum. The IS warfarin was detected in FS using the $[M+H]^+$ at *m/z*: 309.1121. Analysis of data was performed with XCalibur software. (a) was used for mouse plasma stability, while (b) was used for human plasma stability.

NMR spectroscopy

NMR spectra were recorded on a Bruker Avance 500 (^{1}H: 500 MHz), Avance 500HD (^{1}H: 500 MHz,) or Avance 600 (^{1}H: 600 MHz) at 298 K. Chemical shifts (δ) are reported in parts per million (ppm) and referenced to residual nondeuterated solvent signal ($CDCl_3$: ^{1}H: 7.26 ppm; DMSO-d_6: ^{1}H: 2.50 ppm). Coupling constants (J) are reported in Hz with the following abbreviations used to indicate splitting: s = singlet, d = doublet, t = triplet, q = quartet, m = multiplet, br = broad signal. The R, G, D, f, K refer to the one letter code of amino acids.

Synthetic procedures

Synthesis of alkyne-functionalized PEG5-linker (19)

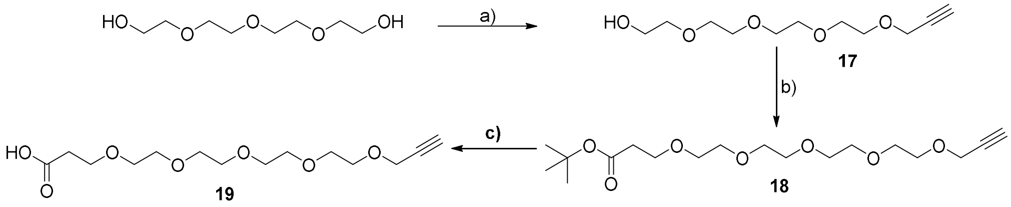

Scheme SI. Synthesis of alkyne-functionalized PEG5-linker (**19**). Reagents and conditions: a) NaH, propargyl bromide, THF, RT, o.n.; b) KO^{t}Bu, ^{t}Bu-acrylate, THF, RT, o.n.; c) TFA/H_2O in DCM, RT, 2.5 h.

3,6,9,12-tetraoxapentadec-14-yn-1-ol (17)

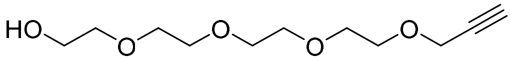

Tetraethylene glycol (12.052 g, 62 mmol, 1 eq) was dissolved in dry THF (22 mL). Sodium hydride (1.036 g, 43 mmol, 0.7 eq) was added at 0 °C and the solution was stirred for 30 min. Afterwards propargyl bromide (3.48 mL, 39 mmol, 0.6 eq, 80 w.t.% in toluene) was added dropwise. The solution was stirred overnight at RT. The reaction was quenched with water (150 mL) and was extracted with ethyl acetate (3 × 150 mL). The combined organic layers were washed with brine (150 mL), dried over $MgSO_4$ and concentrated under reduced pressure. The residue was dried in vacuum to afford compound **17** as a yellow oil (3.91 g, 23 mmol, 58% with reference to propargyl bromide).

^{1}H NMR (500 MHz, $CDCl_3$): δ (ppm) = 2.42 (t, J = 2.4 Hz, 1H, -C≡C<u>H</u>), 2.57 (s, 1H, OH), 3.55 – 3.75 (m, 16H, O-<u>CH$_2$-CH$_2$</u>-O), 4.19 (d, J = 2.3 Hz, 2H, -<u>CH$_2$</u>-C≡CH).

Tert-butyl 4,7,10,13,16-pentaoxanonadec-18-ynoate (18)

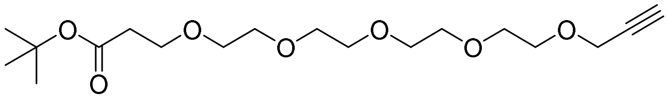

To a solution of KO^{t}Bu (46 mg, 0.4 mmol, 0.05 eq) and **17** (1.74 g, 7.4 mmol, 1 eq) in dry THF (16 mL) ^{t}Bu-acrylate (6 mL, 40 mmol, 5.4 eq) was added dropwise under argon atmosphere and the solution stirred at RT for 22 h. The solvent was evaporated, and the residue was dissolved in water (100 mL). The mixture was extracted with EtOAc (3 × 100 mL), the combined organic layers were washed with brine (50 mL), dried over Na_2SO_4 and the solvent

was removed under reduced pressure. The residue was purified with flash chromatography (PE/EtOAc, 3:2) and **18** (2.0 g, 75%) was obtained as a slightly yellow oil.

^{1}H NMR (500 MHz, $CDCl_3$) δ (ppm)= 1.45 (s, 9H, -OC(CH_3)$_3$), 2.42 (t, J = 2.4 Hz, 1H, -C≡C<u>H</u>), 2.50 (t, J = 6.6 Hz, 2H, O=C-<u>CH$_2$</u>), 3.58 – 3.74 (m, 18H, O-<u>CH$_2$-CH$_2$</u>-O), 4.21 (d, J = 2.4 Hz, 2H, -<u>CH$_2$</u>-C≡CH).

4,7,10,13,16-pentaoxanonadec-18-ynoic acid (19)

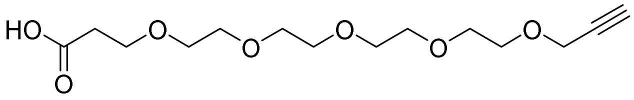

18 (1.42 g, 4.0 mmol) and water (0.92 ml) were dissolved in DCM (20 ml) and TFA (18.4 ml) was added. The reaction was stirred at RT for 2.5 h and the solvents were coevaporated with toluene (3 × 20 mL). **19** (1.35 g, 99%) was obtained as yellow oil.

^{1}H NMR (500 MHz, $CDCl_3$) δ (ppm)= 2.43 (t, J = 2.4 Hz, 1H, -C≡C<u>H</u>), 2.63 (t, J = 6.1 Hz, 2H, -<u>CH$_2$</u>-COOH), 3.61 – 3.81 (m, 18H, O-<u>CH$_2$-CH$_2$</u>-O), 4.20 (d, J = 2.3 Hz, 2H, -<u>CH$_2$</u>-C≡CH).

Synthesis of Alkynyl-PEG5-Val-Cit-PABC-PNP linker (5)

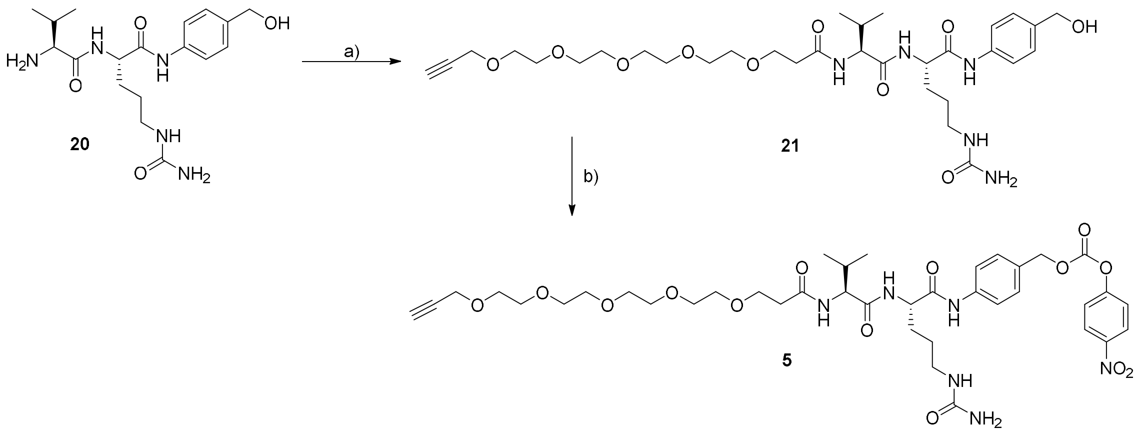

Scheme SII. Synthesis of Alkynyl-PEG5-Val-Cit-PABC-PNP (**5**). Reagents and conditions: a) **19**, HATU, HOAt, DIPEA, DMF, RT, 2 h; b) bis(4-nitrophenyl) carbonate, DIPEA, DMF, RT, 3 h.

Alkynyl-PEG5-Val-Cit-PABOH (21)

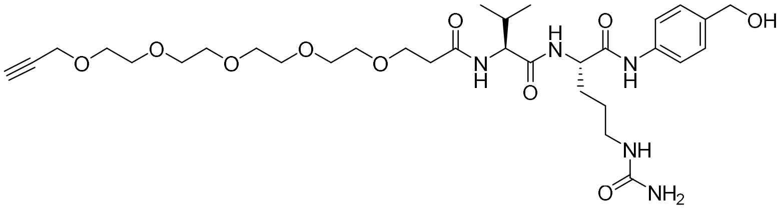

Val-Cit-PABOH (**20**) was synthetized as previously described [1].

20 (103 mg, 0.27 mmol, 1 eq), **19** (100 mg, 0.33 mmol, 1.2 eq), HATU (125 mg, 0.33 mmol, 1.2 eq), HOAt (45 mg, 0.33 mmol, 1.2 eq) and DIPEA (185 µL, 1.09 mmol, 4 eq) were dissolved in DMF (6 mL) and stirred for 2 h at RT. The solvent was removed, the residue was treated with MeOH, sonicated and filtered. The filtrate was concentrated and purified by column chromatography using DCM/MeOH (8:2) as eluent to yield **21** as brownish oil (82.4 mg, 45%).

^{1}H NMR (500 MHz, DMSO-d$_6$): δ (ppm) = 0.83 (dd, J = 6.8 Hz, 3H, Val-$^{\gamma}CH_3$), 0.86 (dd, J = 6.8 Hz, 3H, Val-$^{\gamma}CH_3$), 1.34 - 1.48 (m, 2H, Cit-$^{\gamma}CH_2$), 1.59 (m, 1H, Cit-$^{\beta}CH^A\underline{H^B}$), 1.70 (m, 1H, Cit-$^{\beta}C\underline{H^A}H^B$), 1.97 (m, 1H, Val-$^{\beta}$CH), 2.37 (m, 1H, PEG-$^{\alpha}CH^A\underline{H^B}$), 2.47 (m, 1H, PEG-$^{\alpha}C\underline{H^A}H^B$), 2.93 - 3.06 (m, 2H, Cit-$^{\delta}CH_2$), 3.40 (t, J = 2.3 Hz, 1H, C≡CH), 3.44 - 3.56 (m, 16H, PEG-CH_2), 3.60 (m, 2H, PEG-$^{\beta}CH_2$), 4.13 (d, J = 2.3 Hz, 1H, ≡C-CH_2), 4.23 (m, 1H, Val-$^{\alpha}$CH), 4.38 (m, 1H, Cit-$^{\alpha}$CH), 4.43 (s, 1H, PAB-$C\underline{H^A}H^B$), 5.37 (s, 1H, PAB-$CH^A\underline{H^B}$), 7.23 (d, J = 8.5 Hz, 1H, PAB-C^{ar}H), 7.40 (d, J = 8.5 Hz, 1H, PAB-C^{ar}H), 7.55 (d, J = 8.5 Hz, 1H, PAB-C^{ar}H), 7.65 (d, J = 8.5 Hz, 1H, PAB-C^{ar}H), 7.87 (d, J = 8.2 Hz, 1H, Cit-NH), 8.13 (dd, J = 7.5 Hz, 1H, Val-NH), 9.89, 10.08 (s, 1H, PAB-NH).

Alkynyl-PEG5-Val-Cit-PABC-PNP (5)

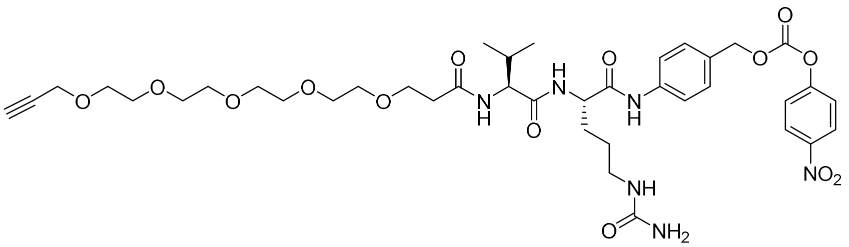

21 (29 mg, 44 µmol, 1 eq) and bis(4-nitrophenyl) carbonate (27 mg, 88 µmol, 2 eq) were dissolved in anhydrous DMF (350 µL) under argon. DIPEA (11 µL, 66 µmol, 1.5 eq) was

added and the mixture was stirred for 3 h at RT. The solvents were removed and RP-HPLC purification (M2/a) yielded **5** as brownish solid (16 mg, 44%).

^{1}H NMR (500 MHz, DMSO-d$_6$): δ (ppm) = 0.83 (dd, J = 6.7 Hz, 3H, Val-$^\gamma$CH$_3$), 0.87 (dd, J = 6.7 Hz, 3H, Val-$^\gamma$CH$_3$), 1.34 - 1.48 (m, 2H, Cit-$^\gamma$CH$_2$), 1.60 (m, 1H, Cit-$^\beta$CH^A$\underline{H^B}$), 1.71 (m, 1H, Cit-$^\beta$C$\underline{H^A}$H^B), 1.97 (m, 1H, Val-$^\beta$CH), 2.38 (m, 1H, PEG-$^\alpha$CH^A$\underline{H^B}$), 2.47 (m, 1H, PEG-$^\alpha$C$\underline{H^A}$H^B), 2.92 - 3.06 (m, 2H, Cit-$^\delta$CH$_2$), 3.41 (t, J = 2.4 Hz, 1H, C≡CH), 3.41 - 3.60 (m, 16H, PEG-CH$_2$), 3.60 (m, 2H, PEG-$^\beta$CH$_2$), 4.13 (d, J = 2.4 Hz, 1H, ≡C-CH$_2$), 4.23 (m, 1H, Val-$^\alpha$CH), 4.38 (m, 1H, Cit-$^\alpha$CH), 5.24 (s, 2H, PAB-CH$_2$), 7.41 (d, J = 8.5 Hz, 2H, PAB-C^{ar}H), 7.57 (d, J = 9.1 Hz, 2H, C^{ar}H), 7.65 (d, J = 8.5 Hz, 2H, PAB-C^{ar}H), 7.87 (d, J = 8.6 Hz, 1H, Cit-NH), 8.13 (d, J = 7.4 Hz, 1H, Val-NH), 8.31 (d, J = 9.1 Hz, 2H, C^{ar}H), 10.06 (s, 1H, PAB-NH).

Synthesis of Alkynyl-PEG5-Val-Cit-Gly-Pro linker (6)

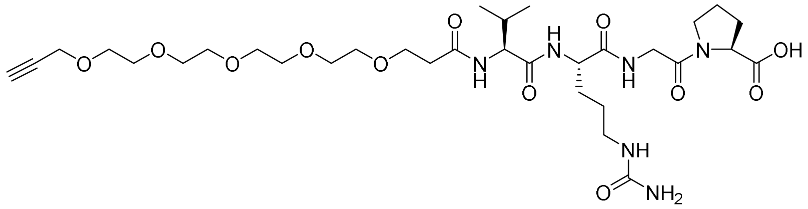

The linear peptide Fmoc-Val-Cit-Gly-Pro-OH was synthesized using Fmoc/^{t}Bu strategy, on 400 mg 2-chlorotrityl chloride resin loaded with 0.92 mmol/g Fmoc-Pro-OH. Elongation of the sequence was performed by sequential Fmoc removal and coupling of the corresponding amino acid. Fmoc-protecting group was removed with 20% piperidine in DMF (2 + 10 min). Coupling of the Fmoc amino acids (4 eq) was performed by treatment with DIC (4 eq) and Oxyma Pure (4 eq) in DMF under stirring at RT for 2 h. **19** (334 mg, 1.1 mmol, 3 eq), Oxyma Pure (156 mg, 1.1 mmol, 3 eq), DIC (170 µL, 1.1 mmol, 3 eq) and DIPEA (375 µL, 2.2 mmol, 6 eq) dissolved in DMF were added to the resin and mixed overnight, finally the resin was washed with DMF and DCM (3 ×).

The resin was treated with 5 mL of 95% TFA, 2.5% H_2O and 2.5% TIS, stirred for 2 h, filtered and washed with TFA (3 mL). The filtrate was concentrated by reduced pressure, the residue was precipitated with cold Et_2O, the crude peptide was decantated, freeze dried and purified by RP-HLPLC (M2/a) to yield **6** as yellow gel (77 mg, 32%).

LC-MS: t_R = 5.81 min, 96% purity (λ =220 nm), *m/z* calcd for $[C_{32}H_{55}N_6O_{12}]^+$: 715.39 $[M + H]^+$, found: 715.40.

Synthesis of azido-functionalized RGD-ligand (9)

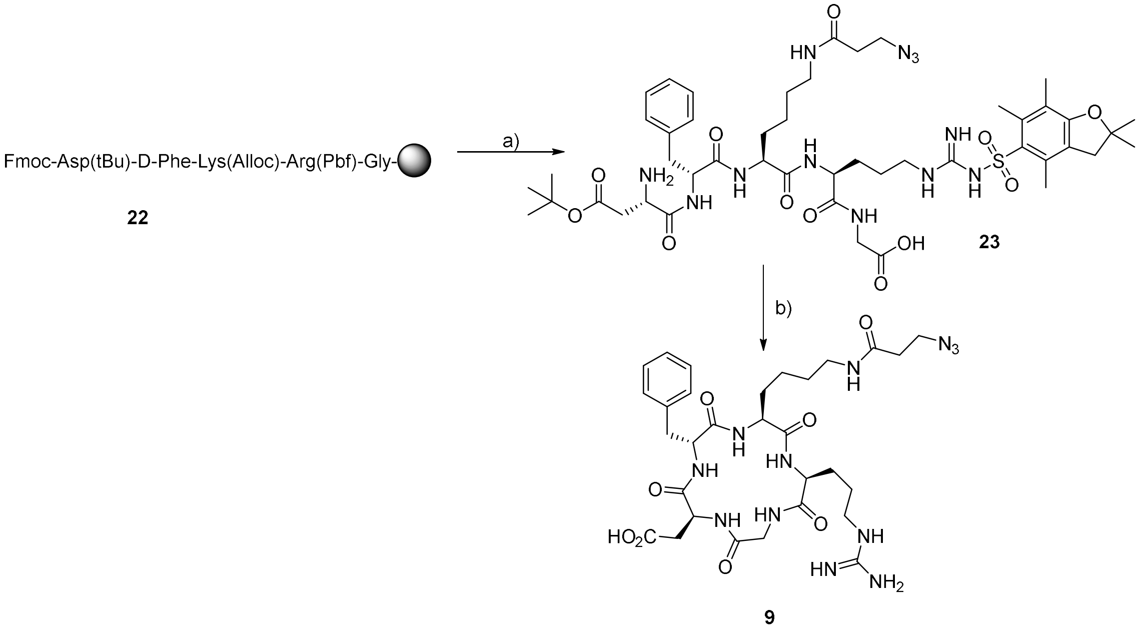

Scheme SIII. Synthesis of azido-functionalized RGD-ligand (**9**). Reagents and conditions: a) 1) Tetrakis(triphenylphosphin)palladium(0), morpholine, DCM, RT, 8 min, 2) 3-azidopropinoic acid, Oxyma Pure, DIC, DMF, RT, 16 h; 3) 20% piperidine/DMF, 4) HFIP/DCM 2 x 3 min; b) 1) HOAt, HATU, DIPEA, DMF, RT, 16 h, 2) TFA/TIS/H_2O, RT, 2 h.

Linear Fmoc-Asp(tBu)-D-Phe-Lys(Alloc)-Arg(Pbf)-Gly-OH peptide (22)

The linear peptide Fmoc-Asp(tBu)-D-Phe-Lys(Alloc)-Arg(Pbf)-Gly-OH was synthesised using Fmoc/^{t}Bu strategy, on 250 mg 2-chlorotrityl chloride resin loaded with 0.96 mmol/g Fmoc-Gly-OH.

Linear H-Asp(^{t}Bu)-D-Phe-Lys(-CO-CH$_2$-CH$_2$-N$_3$)-Arg(Pbf)-Gly-OH (23)

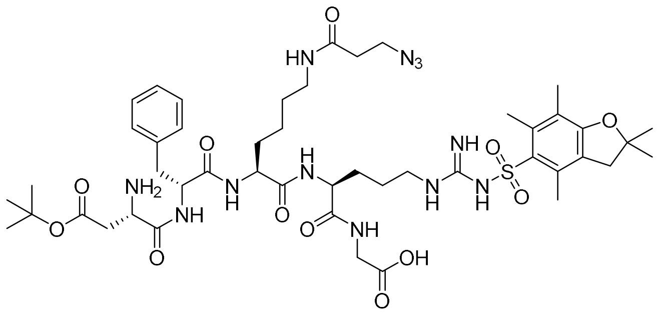

The resin was loaded in a syringe and washed with DCM, DMF and Et$_2$O. For Alloc deprotection, Tetrakis(triphenylphosphin)palladium(0) (88.5 mg, 0.07 mmol, 0.3 eq) and morpholine (221 µL, 2.55 mmol, 10 eq) suspended in dry DCM (1 mL) were added to the resin under inert atmosphere. After the resin was mixed for 8 minutes, it was rinsed eight times with DCM (5 mL) until the brownish color was removed. The deprotection was repeated one more time and it was monitored by LC-MS upon test-cleavage of the peptide with TFA.

LC-MS: t_R = 6.42 min, *m/z* calcd for $[C_{42}H_{54}N_9O_{10}]^+$: 844.39 $[M + H]^+$, found: 844.42.

After Alloc deprotection, 3-azidopropinoic acid (88 mg, 0.77 mmol, 3eq), Oxyma Pure (109 mg, 0.77 mmol, 3 eq) and DIC (119 µL, 0.77 mmol, 3 eq) dissolved in DMF were added to the resin and mixed overnight, finally the resin was washed with DMF and DCM (3 ×). The terminal Fmoc-protecting group was removed with 20% piperidine in DMF.

The linear azido-functionalized peptide was cleaved from the resin with 25% hexafluroisopropanol/DCM for 2 × 3 minutes. The solvents were removed, the crude peptide was lyophilized, purified by RP-HPLC (M2/a) to obtain **23** as colorless powder (113 mg, 42%).

LC- MS: t_R = 7.27 min, 75% purity (λ =220 nm), *m/z* calcd for $[C_{47}H_{71}N_{12}O_{12}S]^+$: 1027.50 $[M + H]^+$, found: 1027.51.

***Cyclo*(Arg-Gly-Asp-D-Phe-Lys(-CO-CH_2-CH_2-N_3)) (9)**

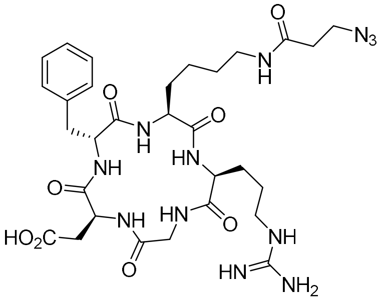

The linear peptide was cyclized as previously described [2]. A solution of HATU (41 mg, 108 µmol, 1 eq) and HOAt (15 mg, 108 µmol, 1 eq) in 5 mL DMF and a solution of peptide **23** (113 mg, 108 µmol, 1 eq) in 5 mL DMF were added simultaneously using a dual syringe pump at a flow rate of 0.32 mL/h, to a solution of DMF (29.8 mL, 275 µL/µmol peptide) containing 55.5 µL DIPEA (3 eq). After the addition was completed the reaction was stirred for 2 h, then the solvents were removed, the crude peptide was lyophilized and purified by RP-HLPC (M2/a).

LC-MS: t_R = 9.63 min, *m/z* calcd for $[C_{47}H_{69}N_{12}O_{12}S]^+$: 1009.49 $[M + H]^+$, found: 1009.49.

The cyclic peptide was dissolved in 5 mL of 95% TFA, 2.5% H_2O and 2.5% TIS and stirred for 2 h. The solvents were removed in vacuum, the residue was precipitated with cold Et_2O, the crude peptide was decantated, freeze dried and purified by RP-HLPLC (method M2/a) to yield **9** as colorless powder (11.3 mg, 10 %).

LC-MS: t_R = 5.27 min, >99% purity (λ =220 nm), *m/z* calcd for $[C_{30}H_{45}N_{12}O_8]^+$: 701.34 $[M + H]^+$, found: 701.35.

^{1}H-NMR (600 MHz, DMSO-d_6): δ (ppm) = 1.00-1.05 (m, 2H, K-$^{\gamma}CH_2$), 1.25-1.43 (m, 5H, R-$^{\gamma}CH_2$, K-$^{\delta}CH_2$, K-$^{\beta}CH_2$), 1.44-1.49 (m, 1H, R-$^{\beta}CH_2$), 1.50-1.57 (m, 1H, K-$^{\beta}CH_2$), 1.66-1.71 (m, 1H, R-$^{\beta}CH_2$), 2.36 (t, J = 5.8 Hz, 2H, CH_2-$\underline{CH_2}$-N_3), 2.39 (m, 1H, D-$^{\beta}CH_2$), 2.70 (dd, J = 16.3, 8.6 Hz, 1H, D-$^{\beta}CH_2$), 2.80 (dd, J = 13.4, 5.9 Hz, 1H, f-$^{\beta}CH_2$), 2.92 (dd, J = 13.4, 8.4 Hz, 1H, f-$^{\beta}CH_2$), 2.97 (ddd, J = 12.7, 7.0, 5.2 Hz, 2H, K-$^{\varepsilon}CH_2$), 3.08 (m, 2H, R-$^{\delta}CH_2$), 3.24 (dd, J = 15.0, 4.2 Hz, 1H, G-$^{\alpha}CH_2$), 3.50 (t, J = 6.4 Hz, 2H, $\underline{CH_2}$-CH_2-N_3), 3.91 (ddd, J = 10.1, 7.4, 4.6 Hz, 1H, K-$^{\alpha}CH$), 4.03 (dd, J = 15.0, 7.6 Hz, 1H, G-$^{\alpha}CH_2$), 4.14 (ddd, J = 8.1, 8.1, 6.1 Hz, 1H, R-$^{\alpha}CH$), 4.43 (ddd, J = 7.3, 7.3, 7.3 Hz, 1H, f-$^{\alpha}CH$), 4.63 (ddd, J = 8.5, 8.5, 5.8 Hz, 1H, D-$^{\alpha}CH$), 7.15 (d, J = 6.9 Hz, 2H, Ph-*orto*), 7.18 (t, J = 7.3 Hz, 1H, Ph-*para*), 7.26 (dd, J = 7.5, 7.5 Hz, 2H, Ph-*meta*), 7.49 (dd, J = 5.9, 5.9 Hz, 1H, R-$^{\delta}NH$), 7.60 (td, J = 7.9 Hz, 1H, R-NH),

7.96 (dd, J = 5.6, 5.6 Hz, 1H, K-$^{\varepsilon}$NH), 8.01 (d, J = 7.2 Hz, 1H, f-NH), 8.04 (d, J = 7.3 Hz, 1H, K-NH), 8.08 (d, J = 8.5 Hz, 1H, D-NH), 8.41 (dd, J = 7.6, 4.4 Hz, 1H, G-NH), 12.24 (s, 1H, D-COOH).

Synthesis of Cy5.5 labeled conjugates 15 and 16

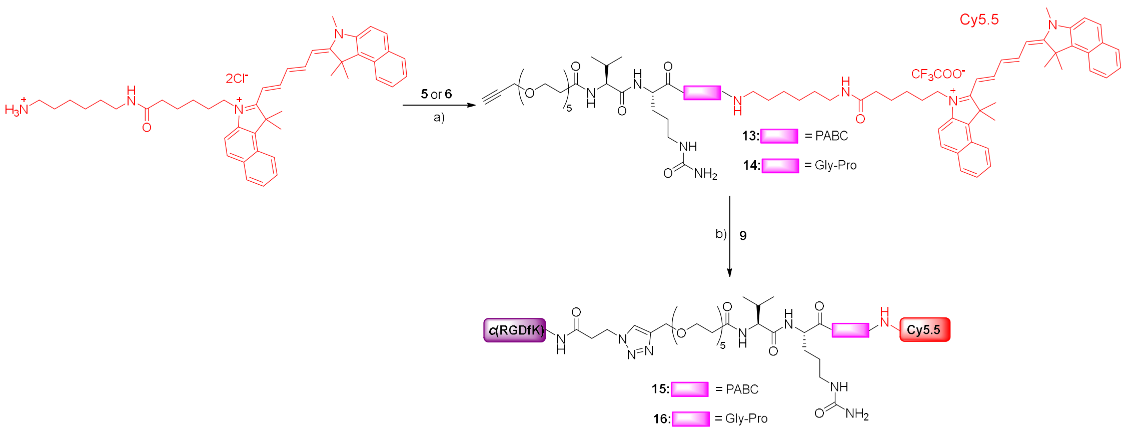

Scheme SIV. Synthesis of conjugates **15** and **16**. Reagents and conditions: a) Cy5.5, **5**, DIPEA, DMF, RT, 5 h or Cy5.5, **6**, PyBOP, HOBt, DIPEA, DMF, RT, 5 h; b) **9**, $CuSO_4 \cdot 5H_2O$, sodium ascorbate, 1:1 DMF/H_2O, 35 °C, 24 h.

Synthesis of Alkynyl-PEG5-Val-Cit-PABC-Cy5.5 (13)

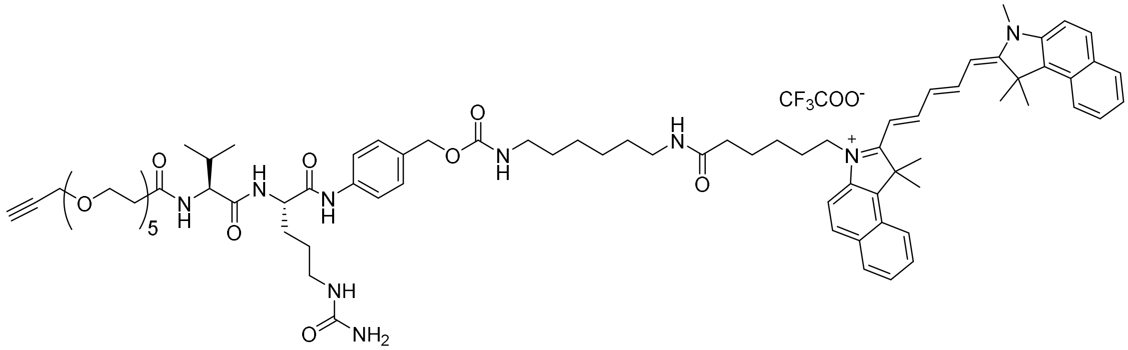

Cy5.5 (5 mg, 6.6 µmol, 1 eq) and **5** (6.6 mg, 7.9 µmol, 1.2 eq) were dissolved in anhydrous DMF (500 µL) under argon. DIPEA (3.4 µL, 29.2 µmol, 3 eq) was added and the mixture was stirred for 5 h at RT, followed by RP-HPLC purification (method M2/b) to yield **13** as blue solid (6.9 mg, 76%). **LC-MS:** t_R = 9.72 min, 88% purity (λ =220 nm), m/z calcd for $[C_{79}H_{106}N_9O_{12}]^+$: 1372.8 $[M]^+$, found: 1372.80; m/z calcd for $[C_{79}H_{107}N_9O_{12}]^{2+}$: 686.90 $[M+H]^{2+}$, found: 686.90.

Synthesis of Alkynyl-PEG5-Val-Cit-Gly-Pro-Cy5.5 (14)

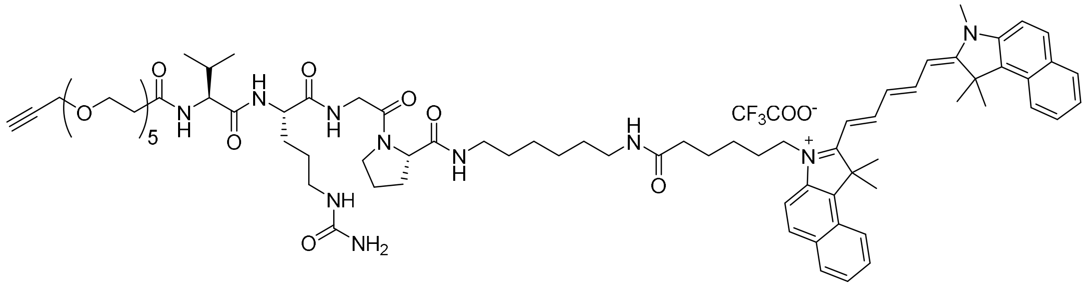

Cy5.5 (5 mg, 6.6 μmol, 1 eq), **6** (19.5 mg, 13.3 μmol, 2 eq), PyBOP (7 mg, 13.3 μmol, 2 eq), HOBt (2.5 mg, 14.9 μmol, 2.25 eq) were dissolved in anhydrous DMF (500 μL) under argon. DIPEA (6 μL, 33.2 μmol, 5 eq) was added and the mixture was stirred for 5 h at RT, followed by RP-HPLC purification (method M2/b) to yield **14** as blue solid (4.0 mg, 43%). **LC-MS:** t_R = 9.33 min, 91% purity (λ =220 nm), *m/z* calcd for $[C_{78}H_{109}N_{10}O_{12}]^+$: 1377.82 $[M]^+$, found: 1377.83; *m/z* calcd for $[C_{78}H_{110}N_{10}O_{12}]^{2+}$: 689.41 $[M + H]^{2+}$, found: 689.42.

Synthesis of *c*(RGDfK)-PEG5-Val-Cit-PABC-Cy5.5 (15)

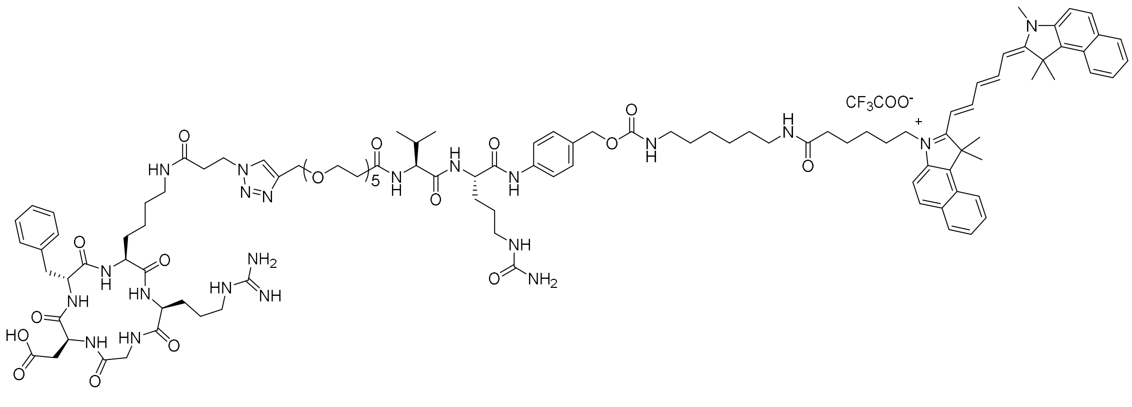

13 (3.3 mg, 2.4 μmol, 1 eq), **9** (1.8 mg, 2.6 μmol, 1.1 eq), $CuSO_4 \cdot 5H_2O$ (0.3 mg, 1.2 μmol, 0.5 eq), sodium ascorbate (0.29 mg, 1.4 μmol, 0.6 eq) were dissolved in DMF/H_2O (1:1, 200 μL, degassed) and stirred for 24 h at 35 °C, followed by RP-HPLC purification (method M2/b) to yield **15** as blue solid (3.1 mg, 62%). **LC-MS:** t_R = 6.97 min, >99% purity (λ =220 nm), *m/z* calcd for $[C_{109}H_{151}N_{21}O_{20}]^{2+}$: 1037.07 $[M + H]^{2+}$, found: 1037.08; *m/z* calcd for $[C_{109}H_{152}N_{21}O_{20}]^{3+}$: 691.72 $[M + 2H]^{3+}$, found: 691.73; *m/z* calcd for $[C_{109}H_{153}N_{21}O_{20}]^{4+}$: 519.04 $[M + 3H]^{4+}$, found: 519.04. **HRMS (ESI-MS)**: *m/z* calcd for *m/z* calcd for $[C_{109}H_{152}N_{21}O_{20}]^{3+}$: 691.7169 $[M + 2H]^{3+}$, found: 691.7147.

Synthesis of *c*(RGDfK)-PEG5-Val-Cit-Gly-Pro-Cy5.5 (16)

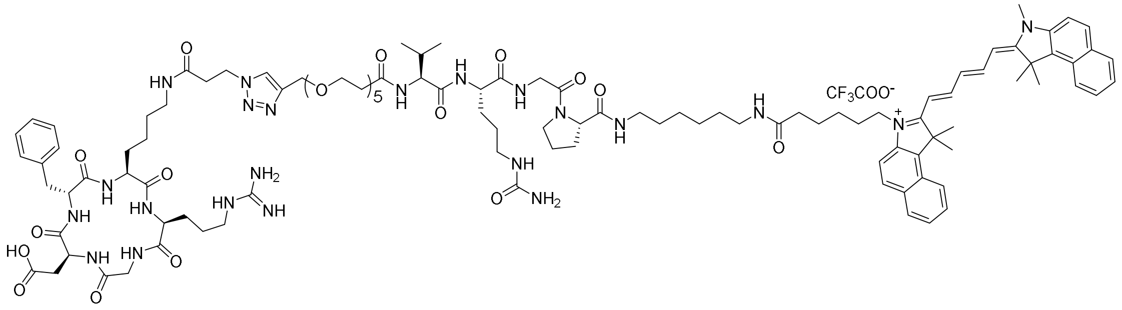

14 (1.5 mg, 1.2 μmol, 1 eq), **9** (0.9 mg, 1.3 μmol, 1.1 eq), $CuSO_4 \cdot 5H_2O$ (0.15 mg, 0.6 μmol, 0.5 eq), sodium ascorbate (0.14 mg, 0.7 μmol, 0.6 eq) were dissolved in DMF/H_2O (1:1, 140 μL, degassed) and stirred for 24 h at 35 °C, followed by RP-HPLC purification to yield **16** as blue solid (1.70 mg, 68%). **LC-MS:** t_R = 6.75 min, >99% purity (λ =220 nm), *m/z* calcd for $[C_{108}H_{154}N_{22}O_{20}]^{2+}$: 1039.58 $[M + H]^{2+}$, found: 1039.59; *m/z* calcd for $[C_{108}H_{155}N_{22}O_{20}]^{3+}$: 693.39 $[M + 2H]^{3+}$, found: 693.42; *m/z* calcd for $[C_{108}H_{156}N_{22}O_{20}]^{4+}$: 520.30 $[M + 3H]^{4+}$, found: 520.30. **HRMS (ESI-MS)**: *m/z* calcd for *m/z* calcd for $[C_{108}H_{155}N_{22}O_{20}]^{3+}$: 693.3924 $[M + 2H]^{3+}$, found: 693.3892.

Characterization details (^{1}H-NMR, HPLC and Mass Spectrometry Data)

17: ^{1}H NMR

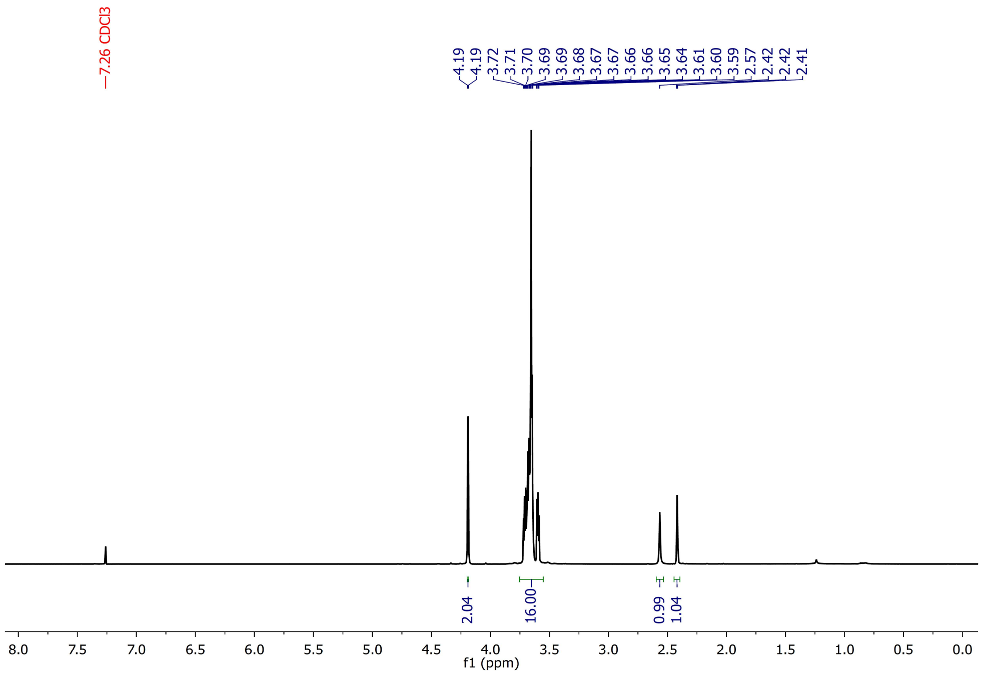

18: ^{1}H NMR

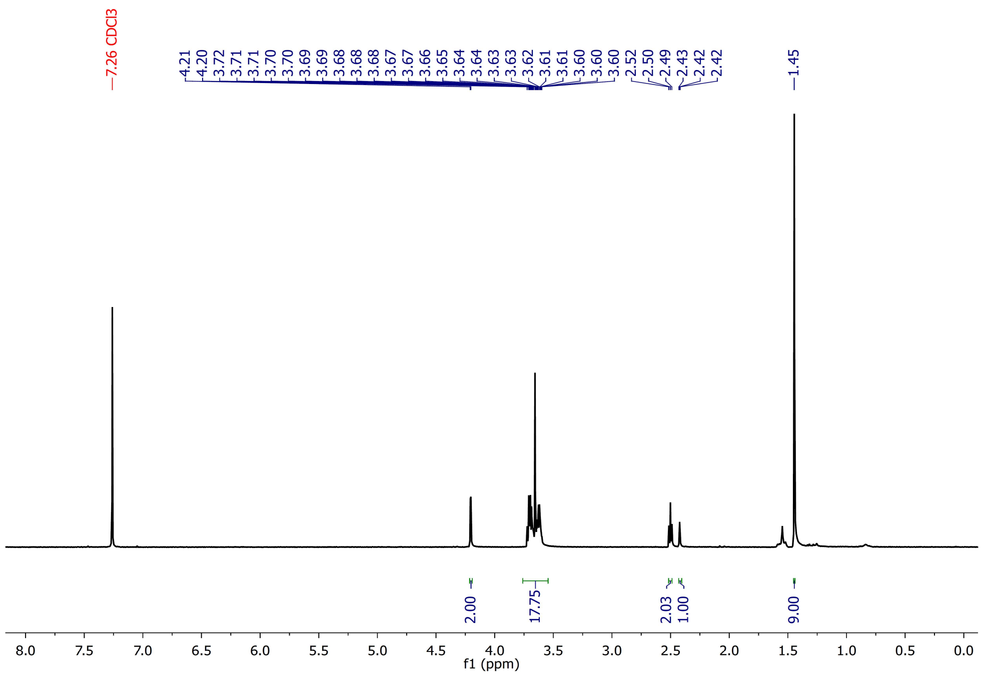

19: ^{1}H NMR

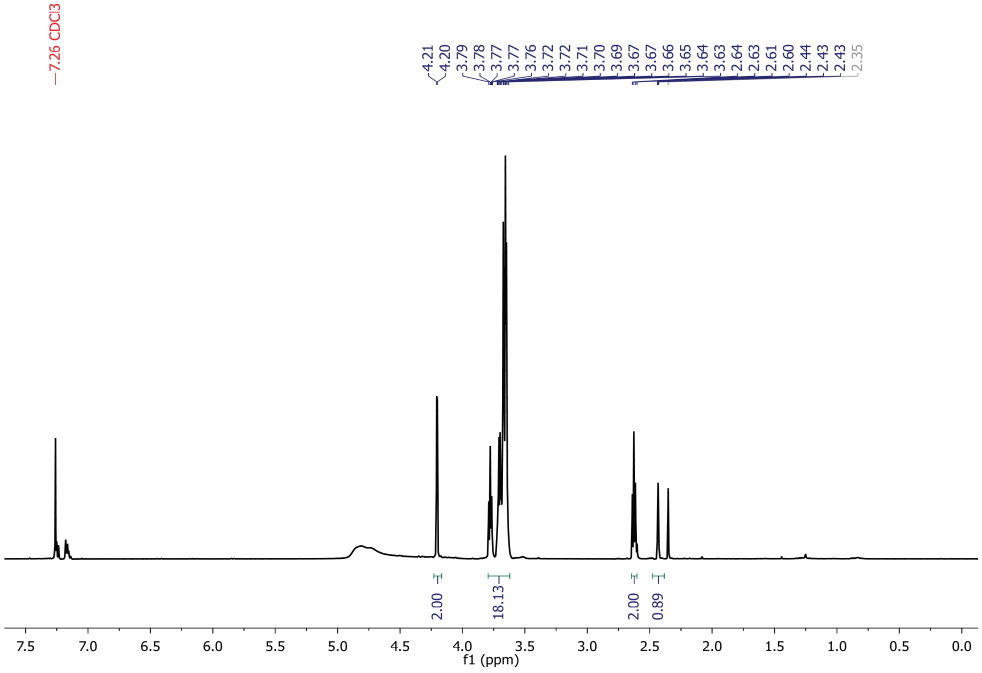

21: ^{1}H NMR

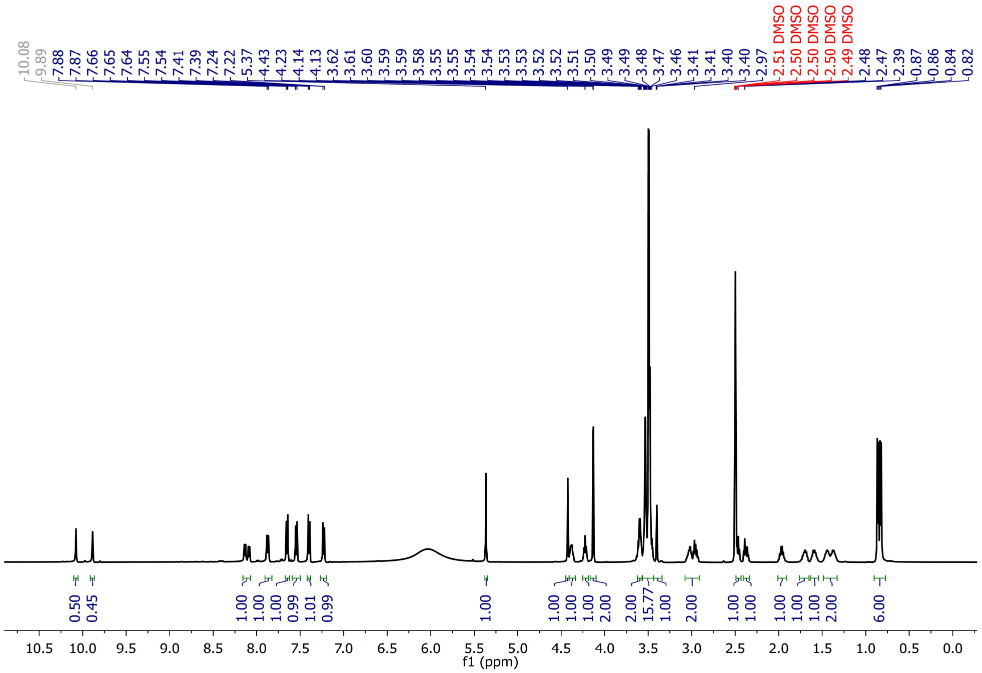

5: ^{1}H NMR

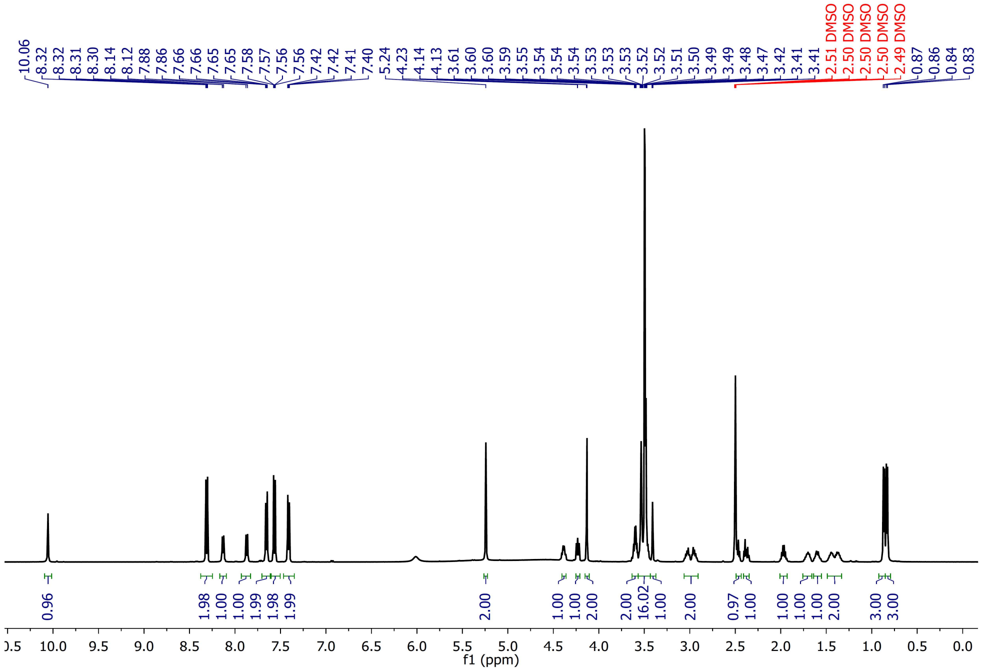

6: HPLC-MS

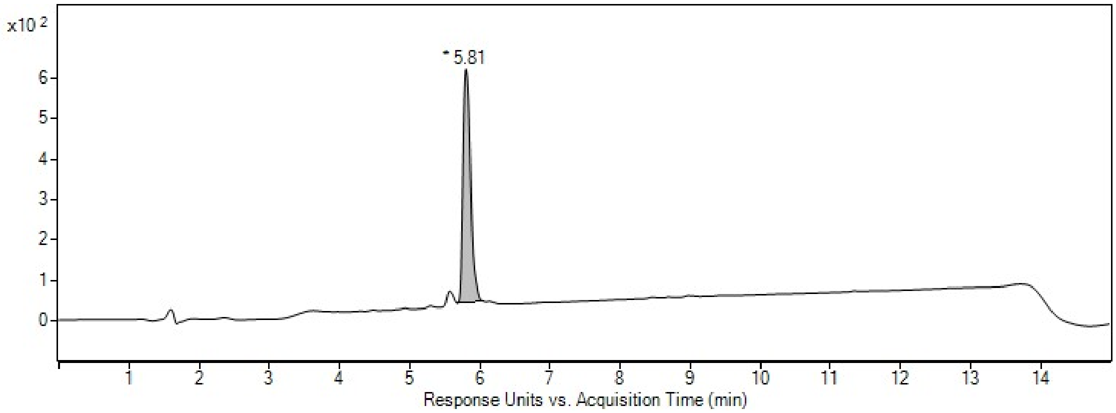
x10 2
6
5
4
3
2
1
0
* 5.81
1
2
3
4
5
6
7
8
9
10
11
12
13
14
Response Units vs. Acquisition Time (min)

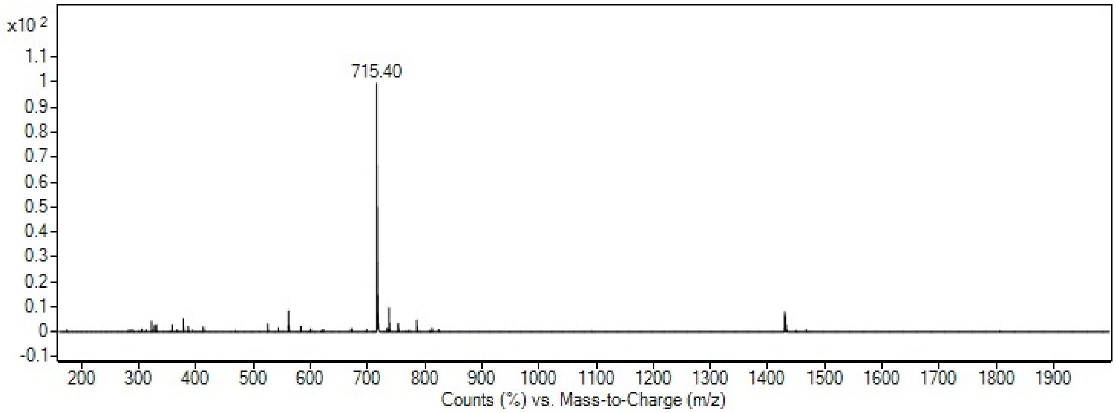
x10 2
1.1
1
0.9
0.8
0.7
0.6
0.5
0.4
0.3
0.2
0.1
0
-0.1
715.40
200
300
400
500
600
700
800
900
1000
1100
1200
1300
1400
1500
1600
1700
1800
1900
Counts (%) vs. Mass-to-Charge (m/z)

23: HPLC-MS

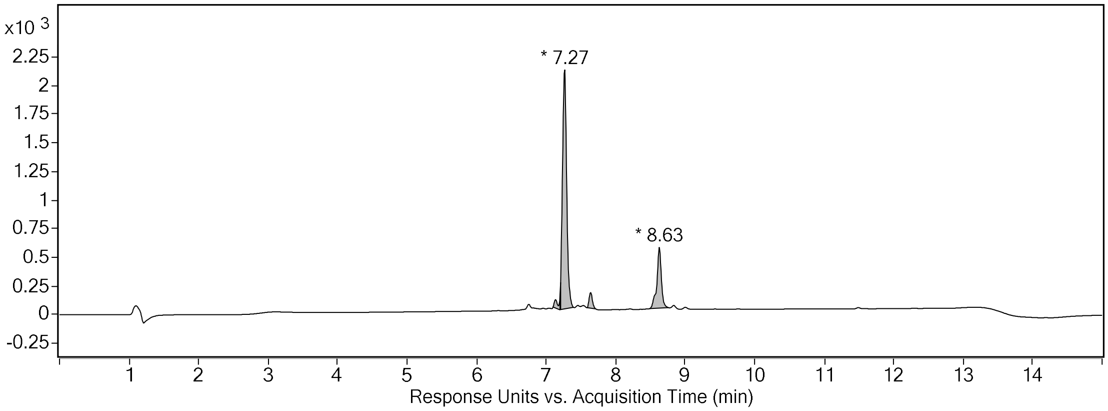

x10 3
* 7.27
* 8.63
Response Units vs. Acquisition Time (min)

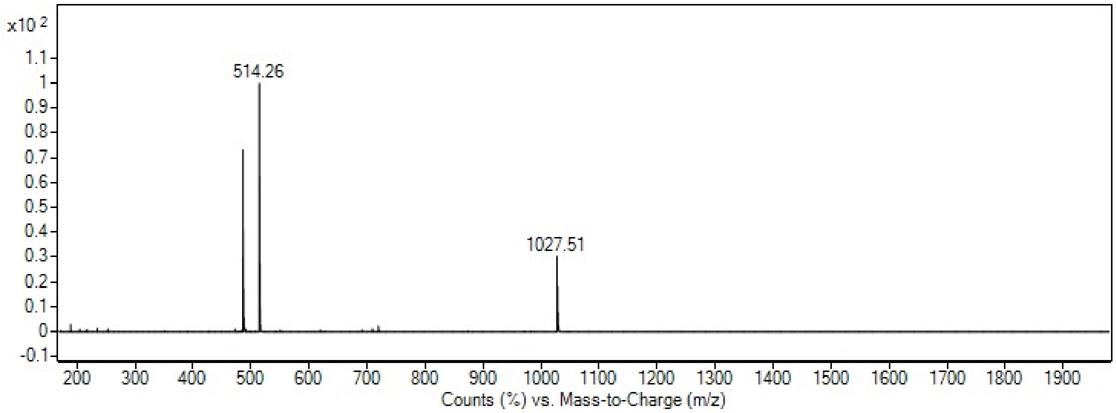

x10 2
514.26
1027.51
Counts (%) vs. Mass-to-Charge (m/z)

9: ^{1}H NMR, ^{1}H - ^{1}H COSY, HPLC-MS

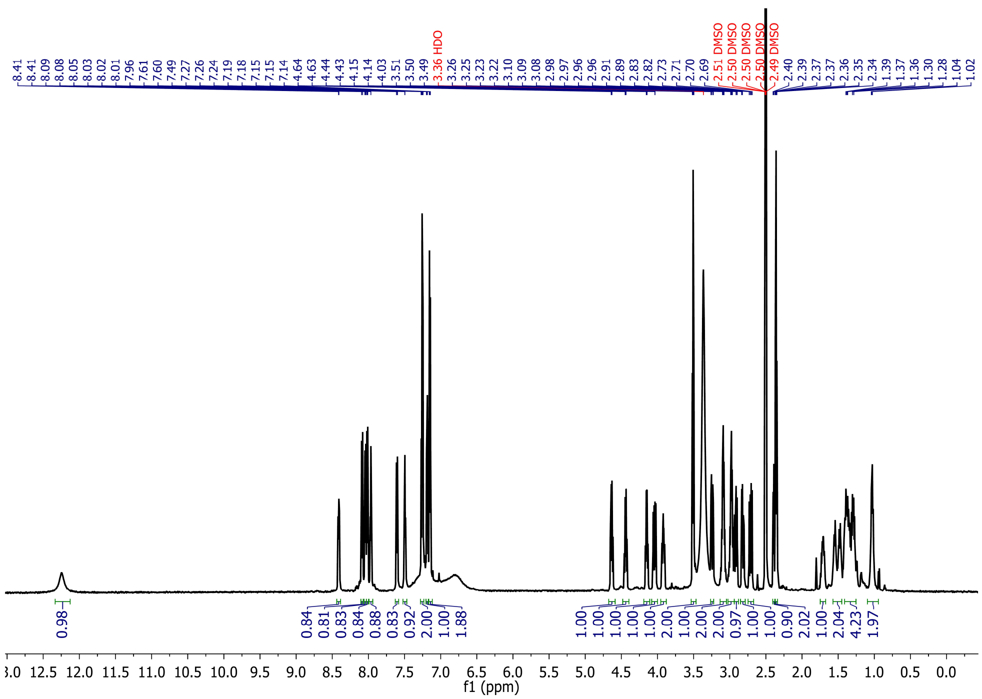

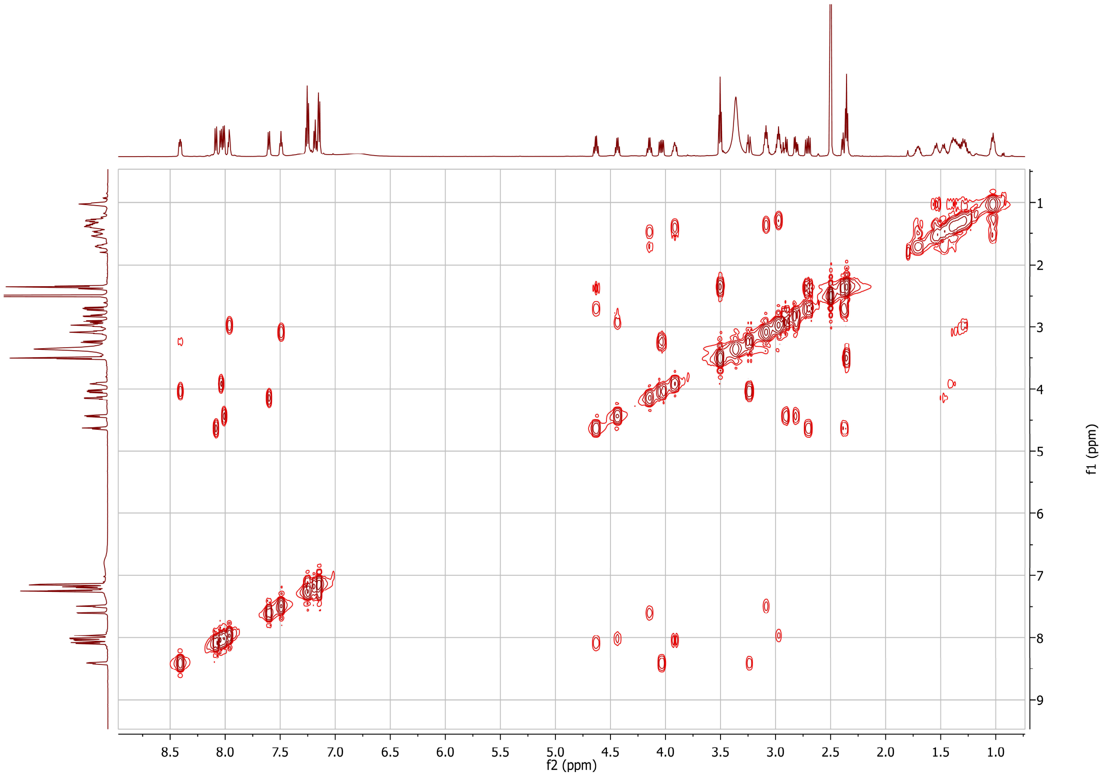

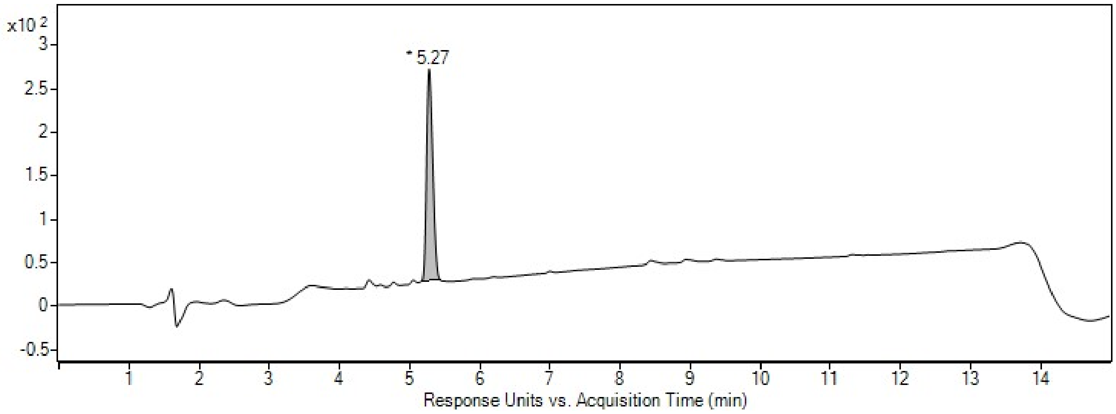
x10 2
* 5.27
Response Units vs. Acquisition Time (min)

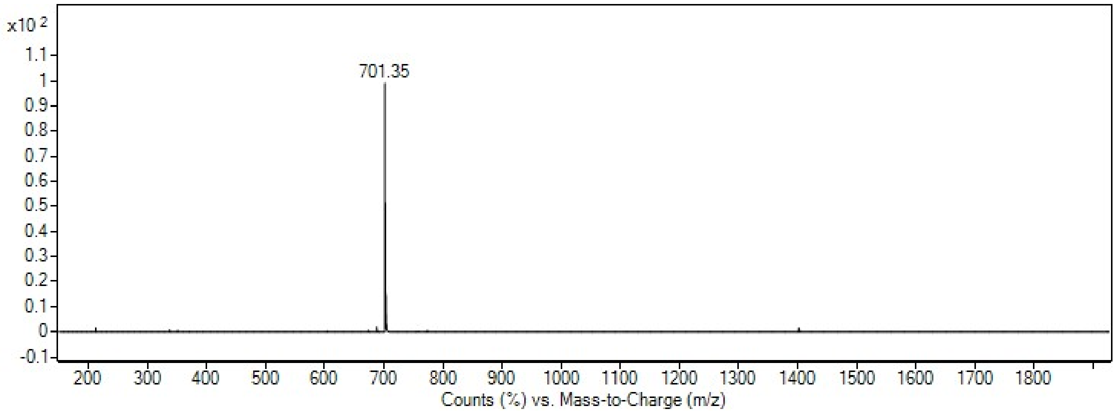
x10 2
701.35
Counts (%) vs. Mass-to-Charge (m/z)

7: HPLC-MS

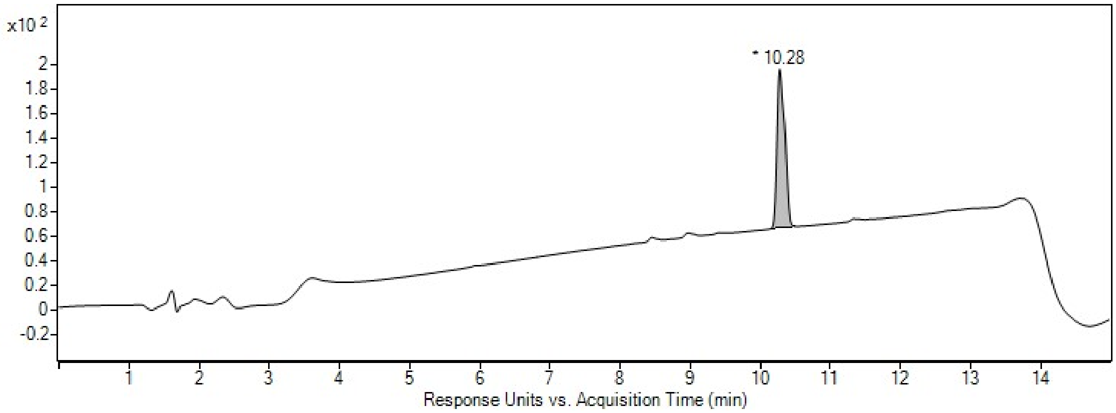
x10 2
* 10.28
Response Units vs. Acquisition Time (min)

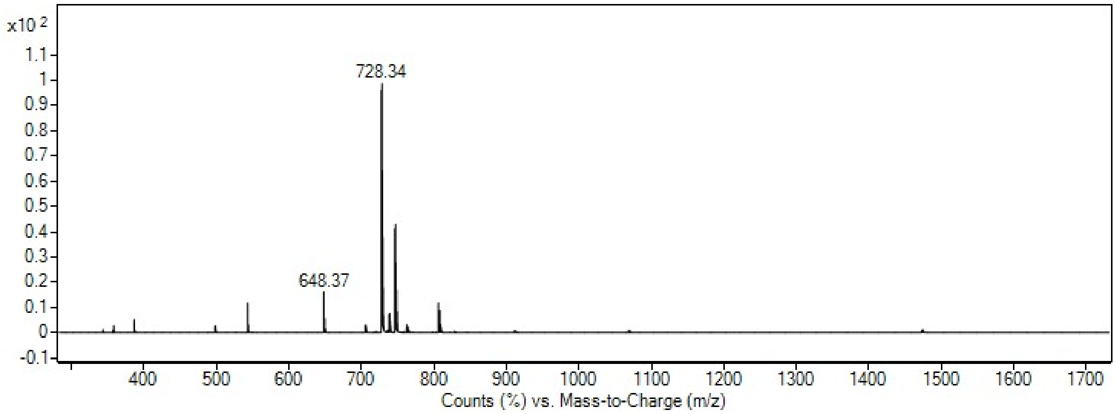
x10 2
728.34
648.37
Counts (%) vs. Mass-to-Charge (m/z)

8: HPLC-MS

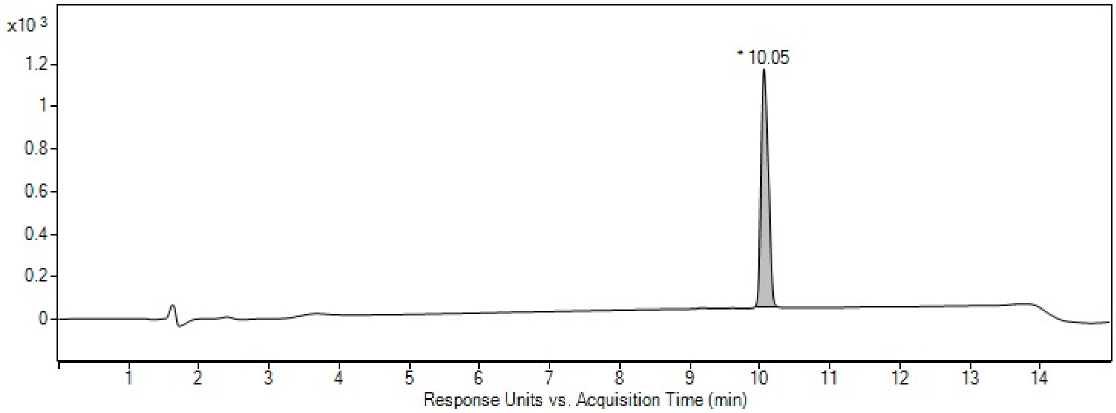

x10 3
* 10.05
Response Units vs. Acquisition Time (min)

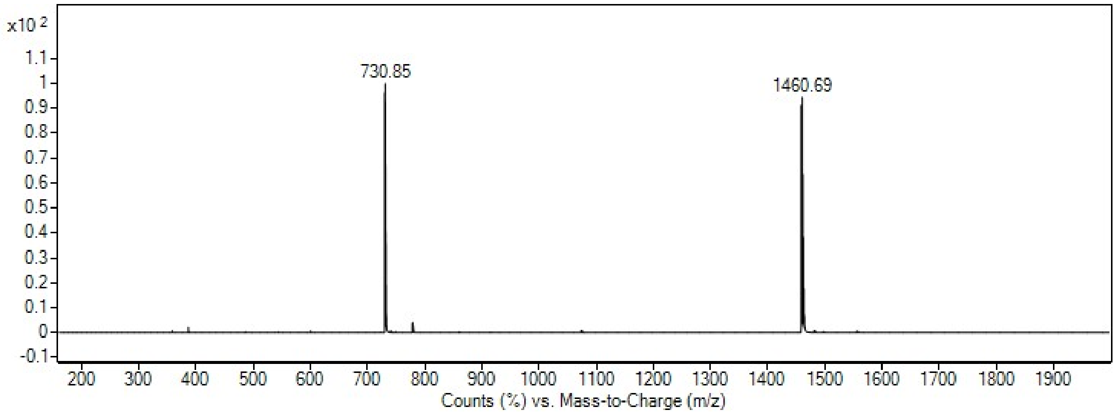

x10 2
730.85
1460.69
Counts (%) vs. Mass-to-Charge (m/z)

10: HPLC-MS, HRMS

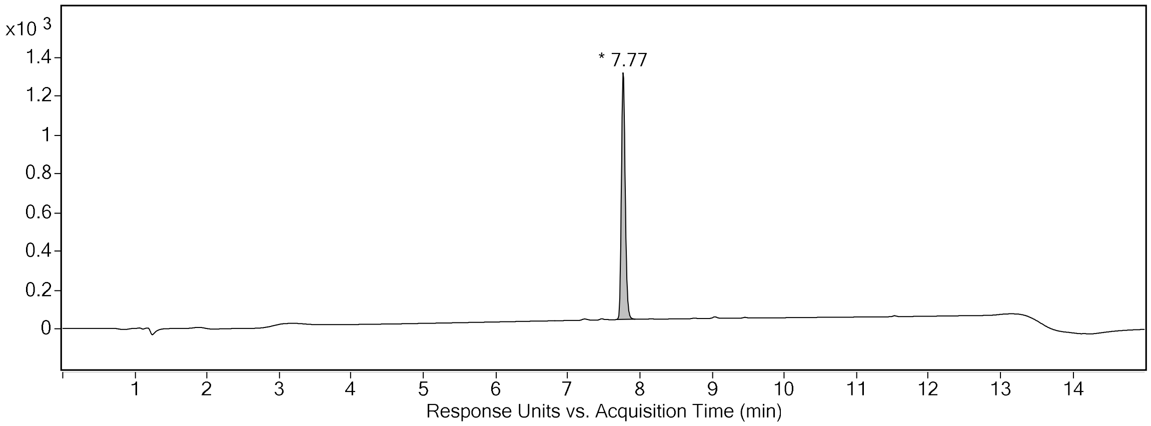

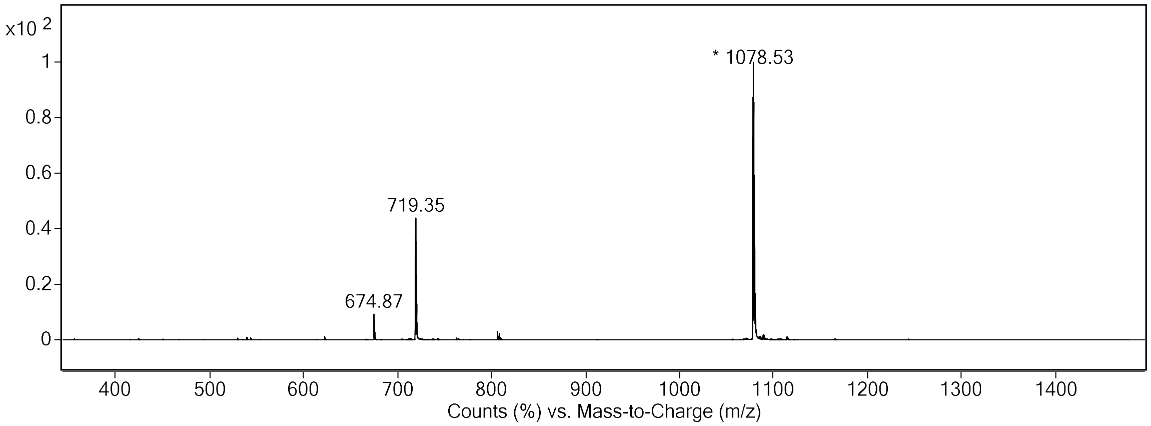

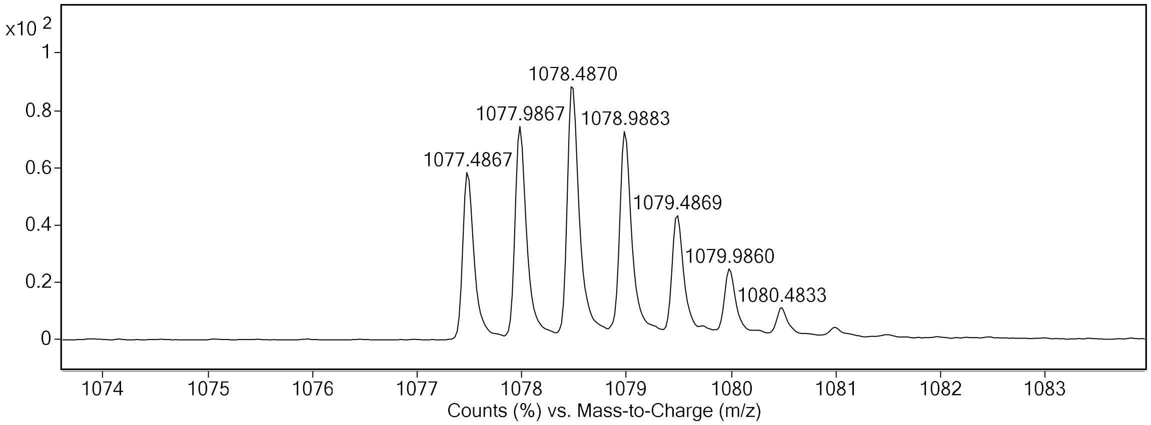

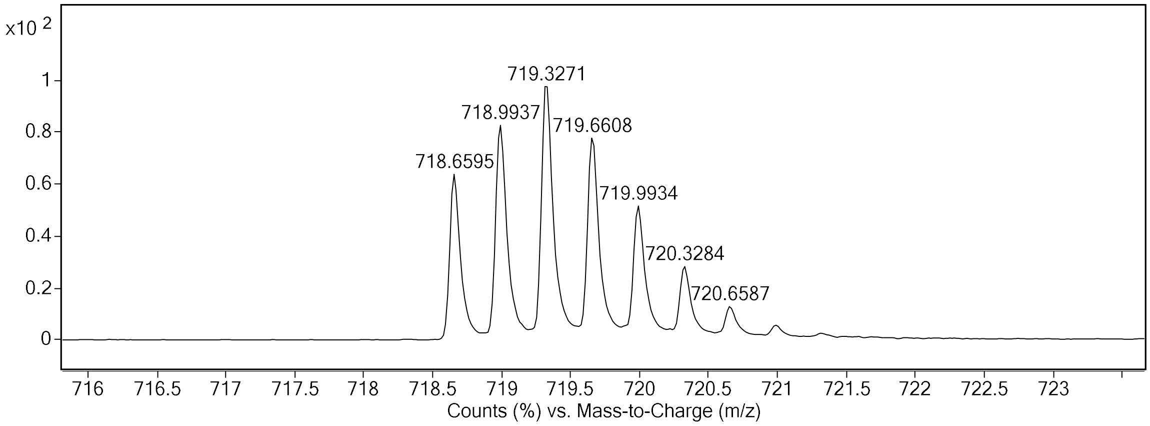
x10 2
1
0.8
0.6
0.4
0.2
0
719.3271
718.9937
719.6608
718.6595
719.9934
720.3284
720.6587
716
716.5
717
717.5
718
718.5
719
719.5
720
720.5
721
721.5
722
722.5
723
Counts (%) vs. Mass-to-Charge (m/z)

11: HPLC-MS, HRMS

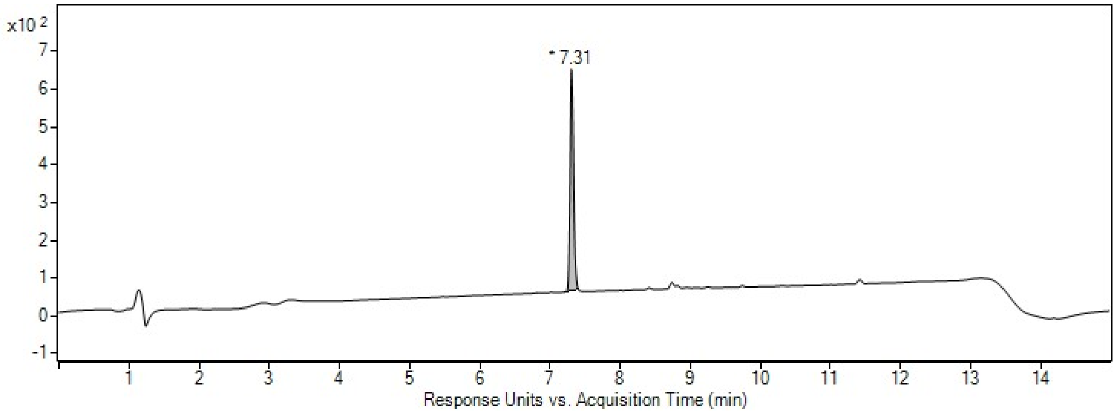

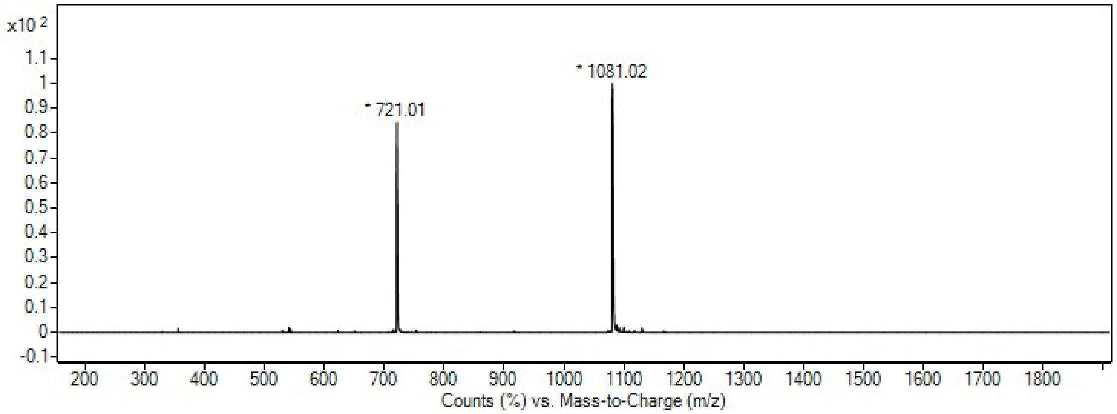

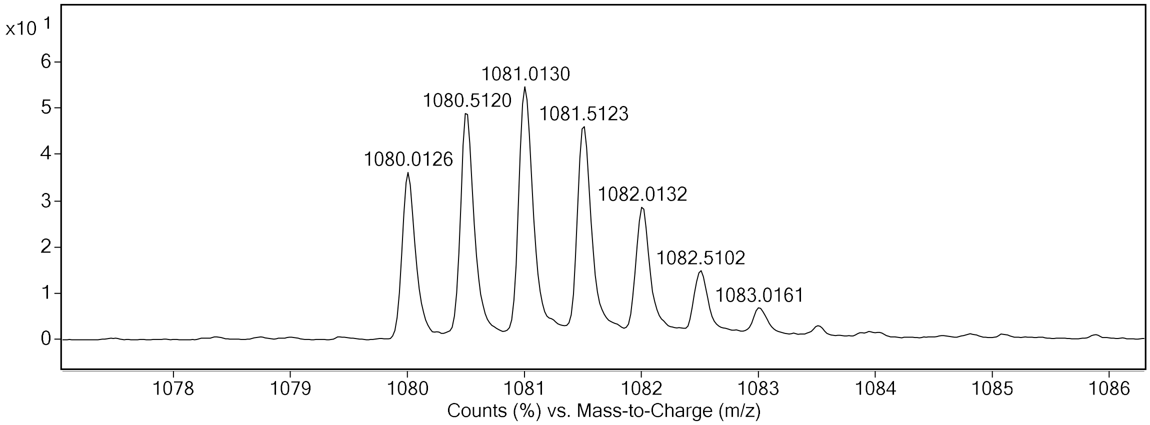

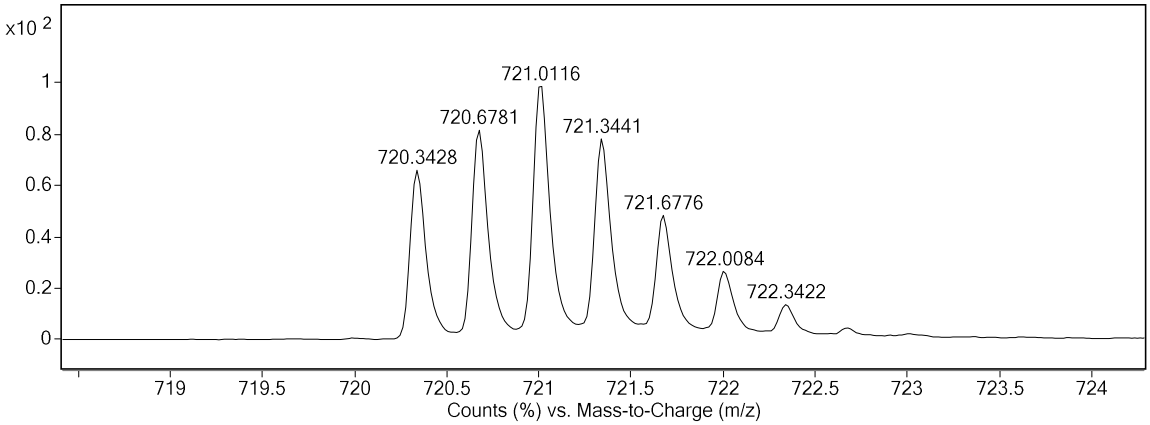

x10 2
1
0.8
0.6
0.4
0.2
0
720.3428
720.6781
721.0116
721.3441
721.6776
722.0084
722.3422
719
719.5
720
720.5
721
721.5
722
722.5
723
723.5
724
Counts (%) vs. Mass-to-Charge (m/z)

13: HPLC-MS

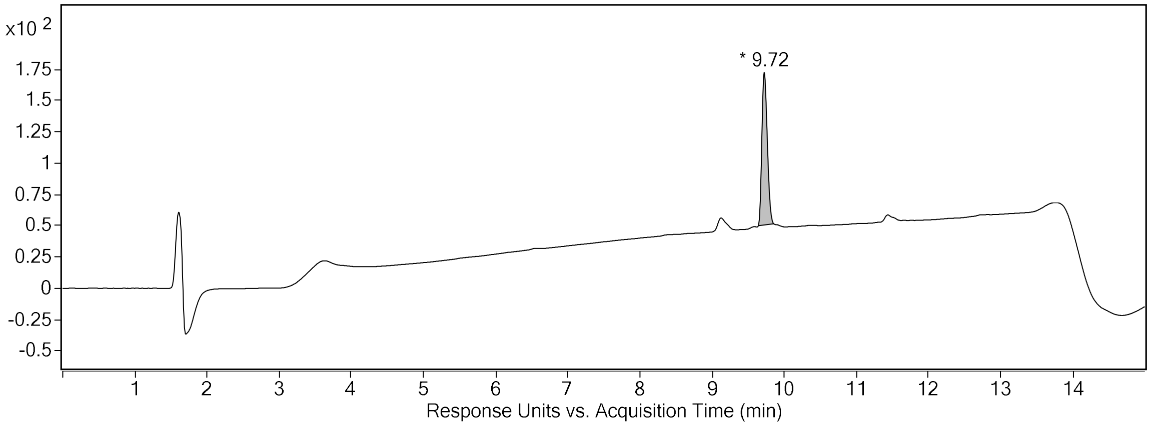
x10 2
* 9.72
Response Units vs. Acquisition Time (min)

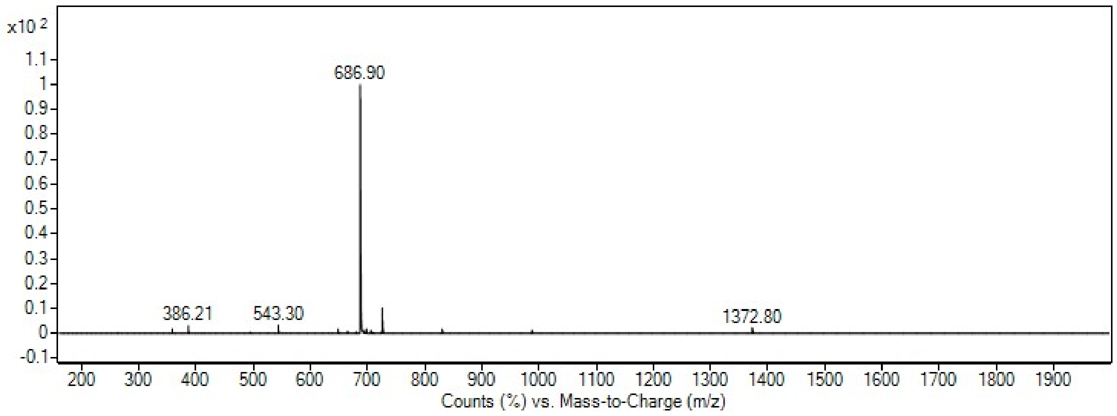
x10 2
686.90
386.21
543.30
1372.80
Counts (%) vs. Mass-to-Charge (m/z)

14: HPLC-MS

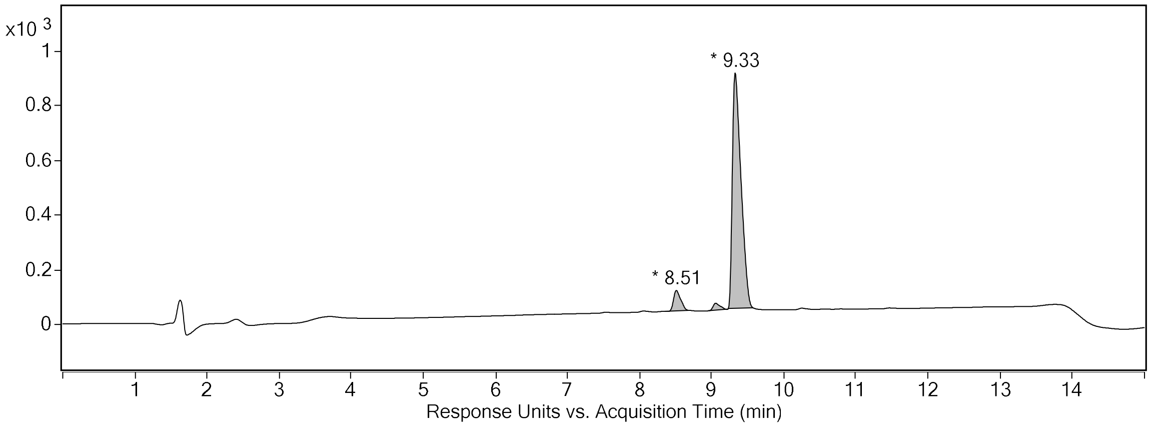
x10 3
1
0.8
0.6
0.4
0.2
0
* 9.33
* 8.51
1 2 3 4 5 6 7 8 9 10 11 12 13 14
Response Units vs. Acquisition Time (min)

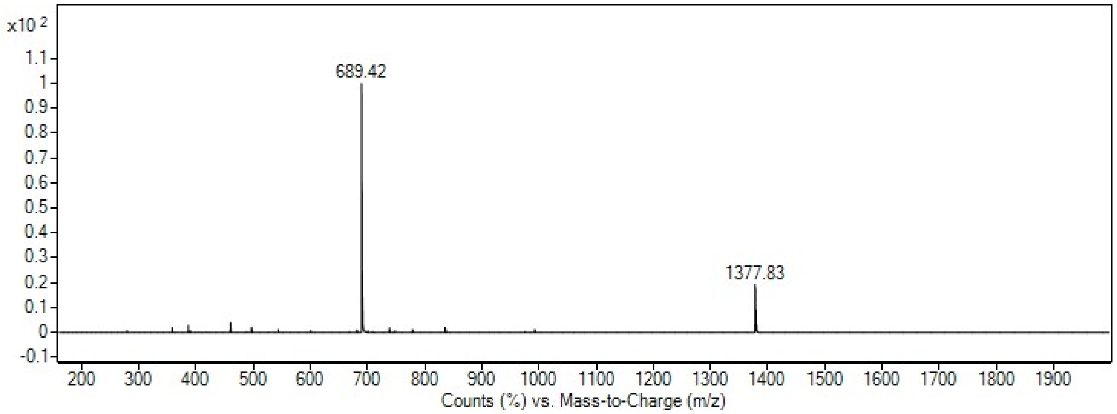
x10 2
1.1
1
0.9
0.8
0.7
0.6
0.5
0.4
0.3
0.2
0.1
0
-0.1
689.42
1377.83
200 300 400 500 600 700 800 900 1000 1100 1200 1300 1400 1500 1600 1700 1800 1900
Counts (%) vs. Mass-to-Charge (m/z)

15: HPLC-MS, HRMS

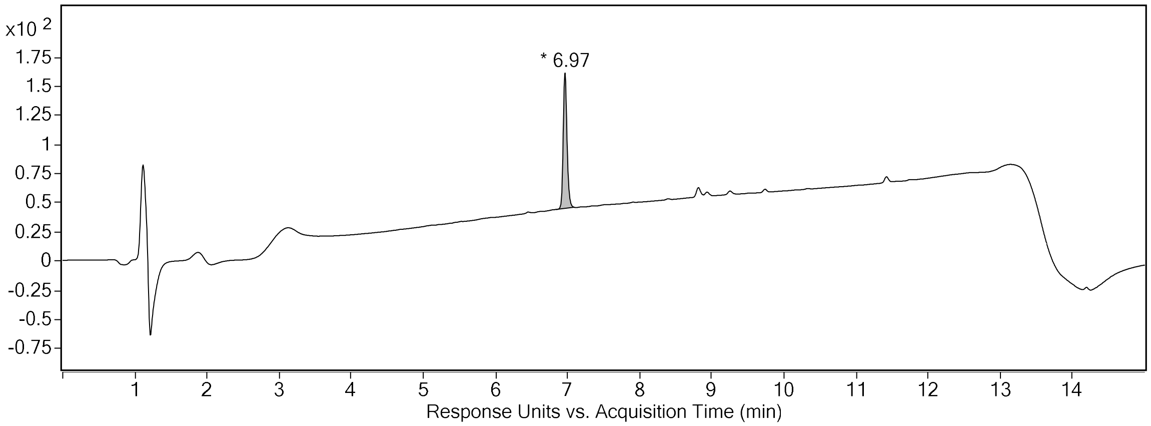

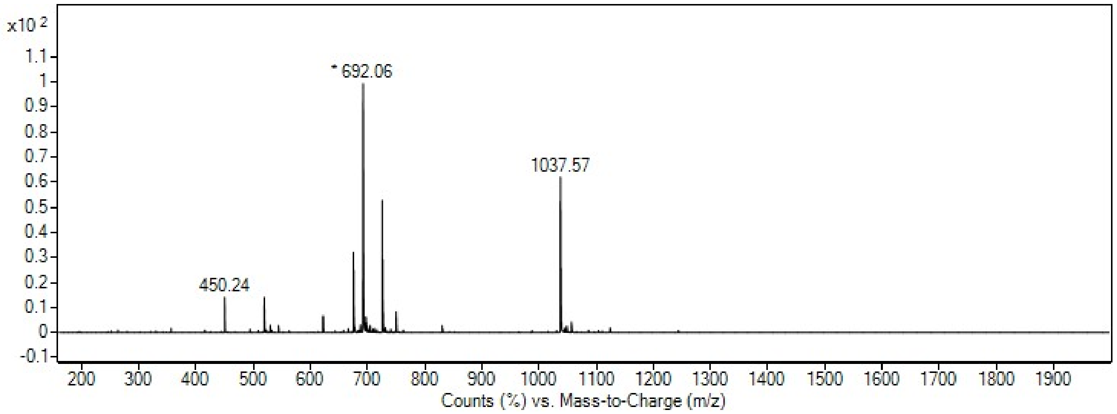

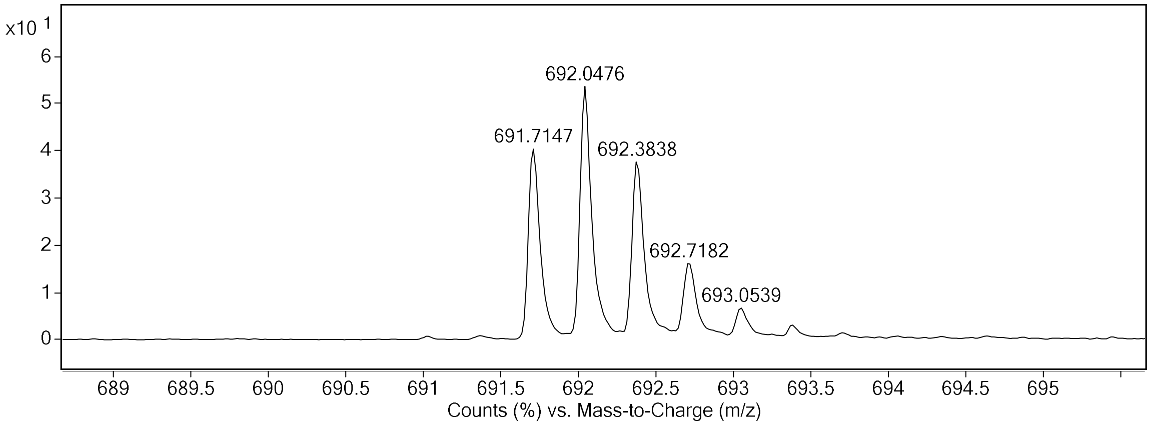

16: HPLC-MS, HRMS

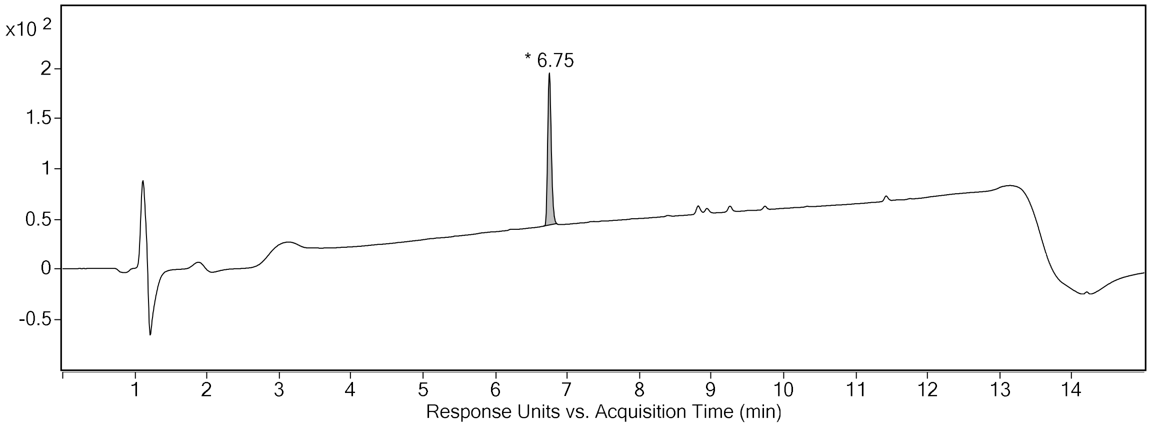

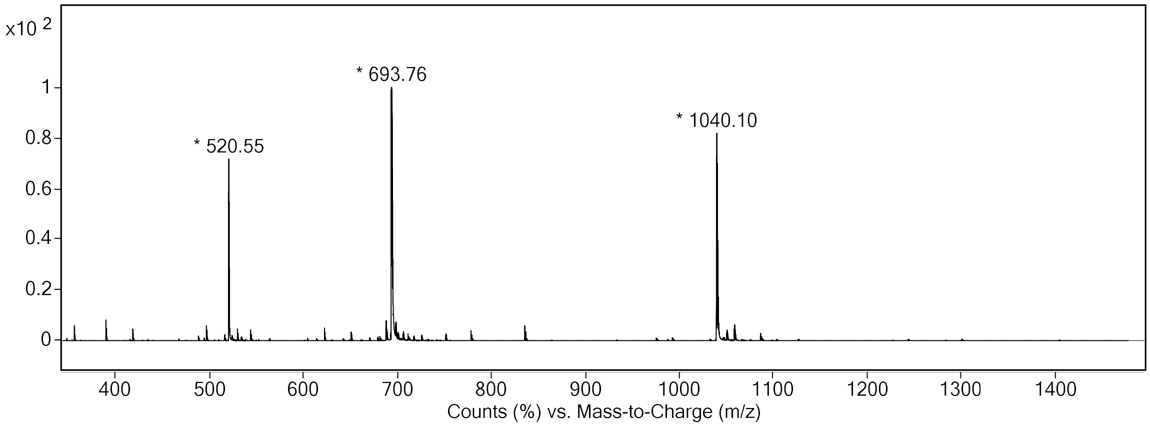

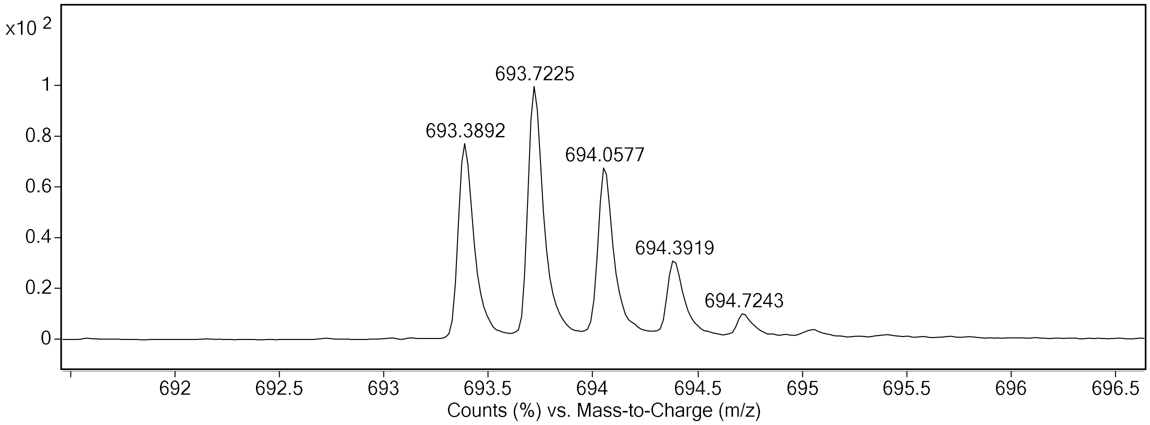

Supplementary figures

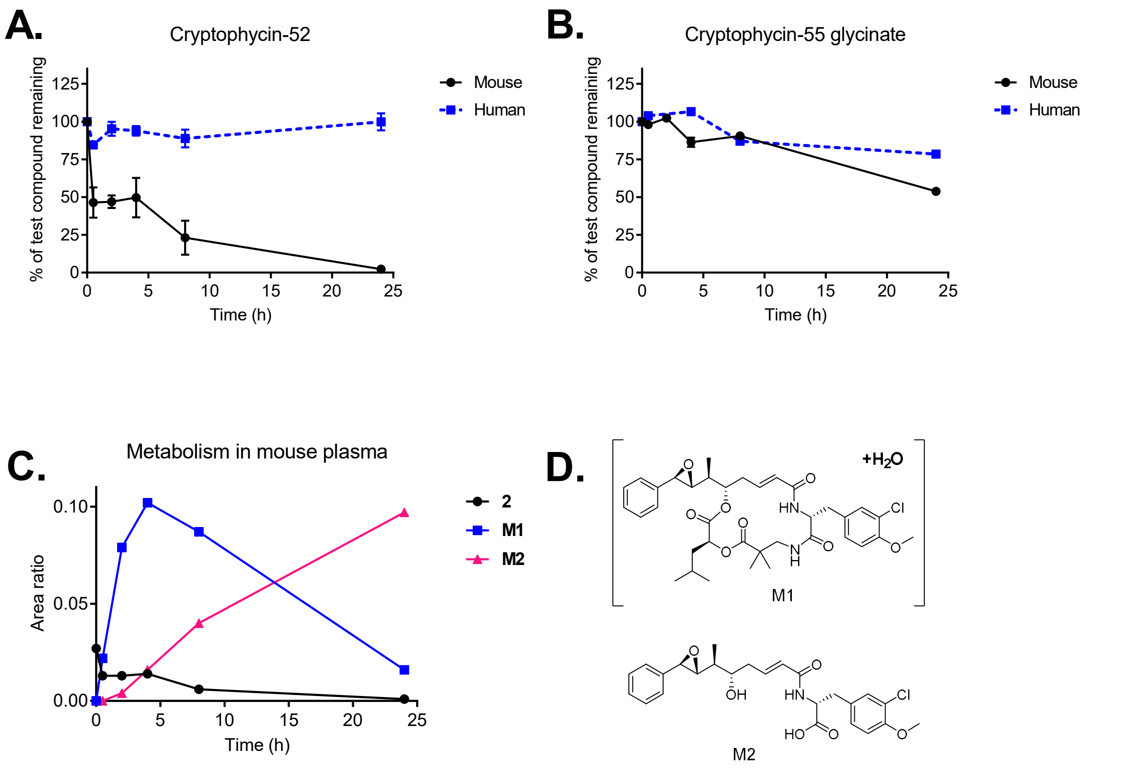

Figure S1. Plasma stability of cryptophycin-52 (**A**) and cryptophycin-55 glycinate (**B**). Metabolism of cryptophycin-52 in mouse plasma (**C**) and the structure of the detected metabolites (**D**).

ID	Structure	Formula	Exact mass	m/z	RT	Detected in Mouse plasma	Detected in Human plasma
**10** (parent cmpd)	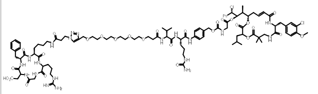	C101H142Cl2N20O28	2152.9680	719.3316	2.99	Detected	Detected
M1	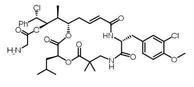	C38H49Cl2N3O9	761.2846	762.2931	2.93	Detected	Detected
M2	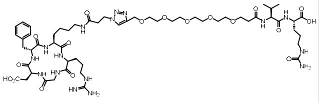	C55H88N16O18	1260.6463	631.3315	1.92	Detected	ND
M3	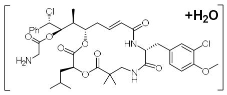	C38H51Cl2N3O10	779.2952	390.6557	2.41	Detected	ND
M4	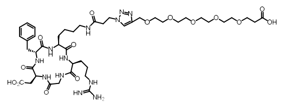	C44H68N12O15	1004.4927	503.2549	1.92	Detected	ND
M5	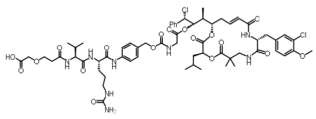	C62H82Cl2N8O18	1296.5124	649.2643	3.44	Detected	ND
M6	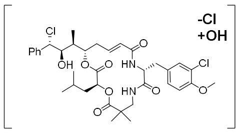	C36H46ClN2O9	686.2970	687.3045	3.42	Detected	Detected

Table S1. Major metabolites of **10** identified after 24 h incubation with mouse and human plasma: proposed structure of metabolites, including also their chemical formula, *m/z*, retention time (RT), and occurrence (ND: not detected).

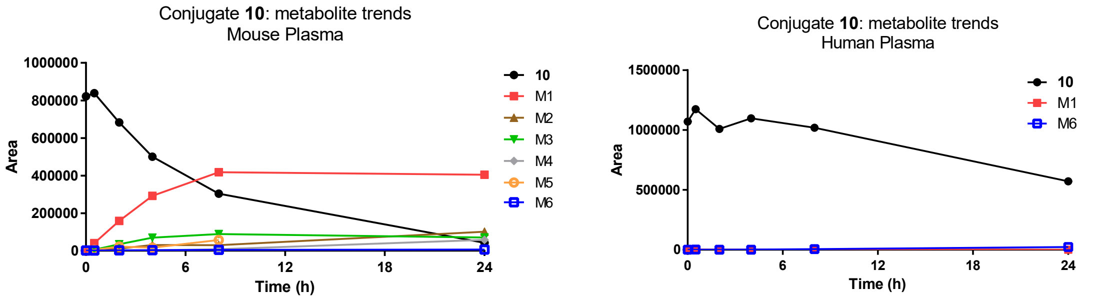

Figure S2. Plot of the compound **10** and metabolites vs. incubation time (h).

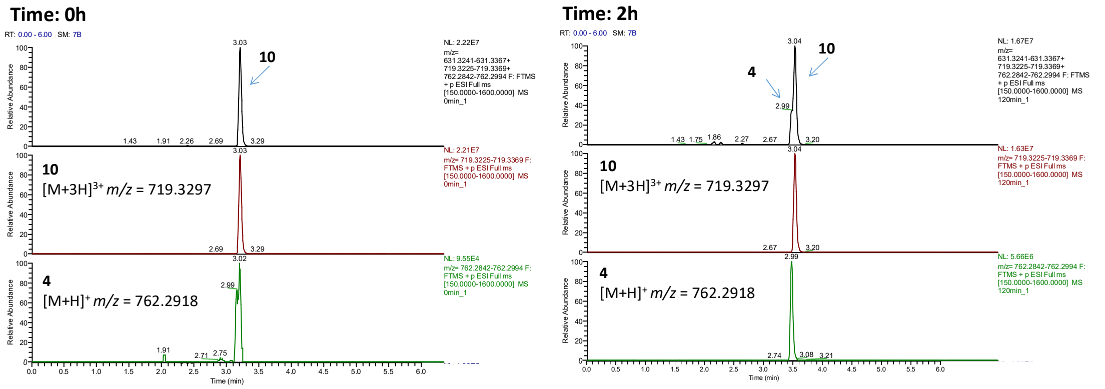

Figure S3. Degradation of conjugate **10** by incubation with lysosomal homogenate for 2 h and the release of cryptophycin-55 glycinate (**4**).

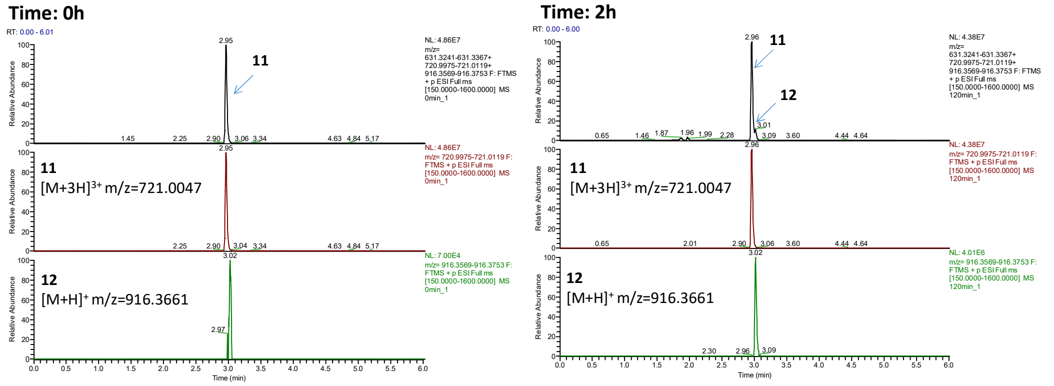

Figure S4. Degradation of conjugate **11** by incubation with lysosomal homogenate for 2 h and the release of Gly-Pro-Cry-55gly (**12**).

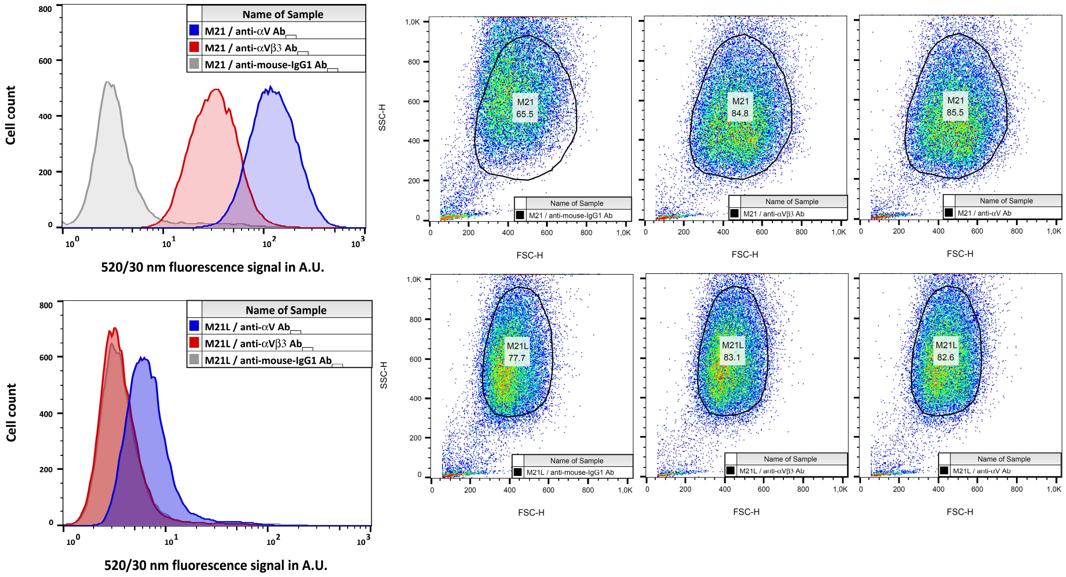

Figure S5. Flow cytometry analysis of integrin α_V and $\alpha_V\beta_3$ expression in M21 and M21-L cell lines.

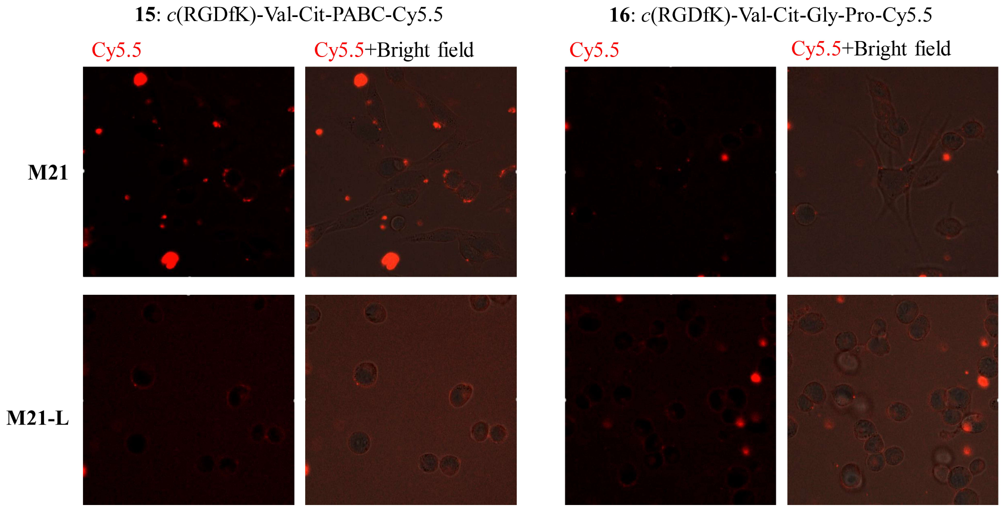

Figure S6. Binding and internalization of compounds **15** and **16** (1μM) in live M21 and M21-L human melanoma cells after incubation at 37 °C for 30 min.

References

(1) Dubowchik, G. M.; Firestone, R. A.; Padilla, L.; Willner, D.; Hofstead, S. J.; Mosure, K.; Knipe, J. O.; Lasch, S. J.; Trail, P. A. Cathepsin B-Labile Dipeptide Linkers for Lysosomal Release of Doxorubicin from Internalizing Immunoconjugates: Model Studies of Enzymatic Drug Release and Antigen-Specific in Vitro Anticancer Activity. *Bioconjug. Chem.* **2002**, *13* (4), 855–869.

(2) Nahrwold, M.; Weiß, C.; Bogner, T.; Mertink, F.; Conradi, J.; Sammet, B.; Palmisano, R.; Royo Gracia, S.; Preuße, T.; Sewald, N. Conjugates of Modified Cryptophycins and RGD-Peptides Enter Target Cells by Endocytosis. *J. Med. Chem.* **2013**, *56* (5), 1853–1864.
